# Molecular Characterization of the Peripheral Airway Field of Cancerization in Lung Adenocarcinoma

**DOI:** 10.1371/journal.pone.0118132

**Published:** 2015-02-23

**Authors:** Jun-Chieh J. Tsay, Zhiguo Li, Ting-An Yie, Feng Wu, Leopoldo Segal, Alissa K. Greenberg, Eric Leibert, Michael D. Weiden, Harvey Pass, John Munger, Alexander Statnikov, Kam-Meng Tchou-Wong, William N. Rom

**Affiliations:** 1 Division of Pulmonary, Critical Care, and Sleep Medicine, Department of Medicine, New York University School of Medicine, New York, New York, United States of America; 2 Center for Health Informatics and Bioinformatics, New York University Langone Medical Center, New York, New York, United States of America; 3 Department of Environmental Medicine, New York University School of Medicine, New York, New York, United States of America; 4 Division of Thoracic Surgery, Department of Cardiothoracic Surgery, New York University School of Medicine, New York, New York, United States of America; 5 Division of Translational Medicine, Department of Medicine, New York University School of Medicine, New York, New York, United States of America; IPMC, CNRS UMR 7275 UNS, FRANCE

## Abstract

*Field of cancerization* in the airway epithelium has been increasingly examined to understand early pathogenesis of non-small cell lung cancer. However, the extent of field of cancerization throughout the lung airways is unclear. Here we sought to determine the differential gene and microRNA expressions associated with field of cancerization in the peripheral airway epithelial cells of patients with lung adenocarcinoma. We obtained peripheral airway brushings from smoker controls (n=13) and from the lung contralateral to the tumor in cancer patients (n=17). We performed gene and microRNA expression profiling on these peripheral airway epithelial cells using Affymetrix GeneChip and TaqMan Array. Integrated gene and microRNA analysis was performed to identify significant molecular pathways. We identified 26 mRNAs and 5 miRNAs that were significantly (FDR <0.1) up-regulated and 38 mRNAs and 12 miRNAs that were significantly down-regulated in the cancer patients when compared to smoker controls. Functional analysis identified differential transcriptomic expressions related to tumorigenesis. Integration of miRNA-mRNA data into interaction network analysis showed modulation of the extracellular signal-regulated kinase/mitogen-activated protein kinase (ERK/MAPK) pathway in the contralateral lung field of cancerization. In conclusion, patients with lung adenocarcinoma have tumor related molecules and pathways in histologically normal appearing peripheral airway epithelial cells, a substantial distance from the tumor itself. This finding can potentially provide new biomarkers for early detection of lung cancer and novel therapeutic targets.

## Introduction

Although lung cancer is the leading cause of cancer deaths worldwide, the early molecular changes remain poorly understood, with more research needed to improve early stage diagnosis and increase survival. The disappointingly low (15%) 5-year survival rate of lung cancer reflects the fact that most patients present with advanced disease [1]. In contrast, when lung cancer is detected in stage I and surgically resected, 10-year survival is as high as 88% [2]. Deciphering the early molecular events in lung tumorigenesis has the potential to improve the clinical management of lung cancer.

Exposure to environmental pollutants, especially cigarette smoke, radon, and asbestos, is the major culprit in initiating and promoting lung tumorigenesis. Exposure to these pollutants and the host inflammatory responses to the irritants result in a histologic and molecular field of injury throughout the central and peripheral airways [3]. The concept of field cancerization, first described by Slaughter et al. in 1953, refers to areas of histologically normal epithelium, adjacent to tumor tissue, but with an abnormal molecular profile similar to that of the tumor itself [4]. In lung cancer, studies have demonstrated that adjacent histologically normal airway epithelial cells have mutations in p53, KRAS, and EGFR genes, aberrant promoter methylation, and allelic loss [5–11]. In addition, studies on microRNAs (miRNAs) and their target messenger RNA transcripts (mRNAs) have shown that these molecules play an important role in lung cancer initiation and progression [12–14]. Differentially expressed miRNAs in normal tissues and lung cancers have been found to target protein-coding tumor suppressors and oncogenes [15,16]. Similarly, gene expression profiles in the proximal airway epithelium of subjects with lung cancer have provided insights into the effect of lung field of cancerization [17].

Although considerable progress has been made in profiling the molecular changes in the field of cancerization, little is known regarding this field effect in the peripheral airway and how far the “field” extends. Transcriptomic studies on peripheral airways of smokers and non-smokers have demonstrated changes in genes coding for immunity, apoptosis, mucin production and response to oxidants and xenobiotics [18,19]. Since adenocarcinoma typically arises from peripheral airway or alveolar epithelial cells, specifically Clara cells and type II pneumocytes, better characterization of the molecular aberrations in these terminal airway bronchoalveolar cells may lead to improved understanding of early events in tumorigenesis. We hypothesized that by using high-throughput technologies to simultaneously profile miRNA and mRNA expression of peripheral airway epithelial cells in high-risk smokers and lung cancer patients, we may, first, find higher-order miRNA-mRNA interactions associated with field of cancerization and tumorigenesis; and second, demonstrate the effect of lung field of cancerization in the contralateral lung of the tumor.

## Methods

### Patient Population

Between April 2010 and May 2012, we recruited 30 subjects (17 with lung adenocarcinoma and 13 smoker controls), and all signed informed consent. Participants included current or former (quit >5 years) smokers (>20 pack-years), age 55–75 years, with suspicious nodules, who were referred for diagnostic bronchoscopy at New York University Langone Medical Center and Bellevue Hospital Center. We excluded those with any previous history of cancer. Seventeen subjects were confirmed to have the diagnosis of adenocarcinoma after the bronchoscopy. Thirteen subjects were classified as control smokers; including smokers with benign nodules after normal diagnostic bronchoscopy and with more than three-year stability on CT scan; and normal smoker volunteers without any pulmonary nodules. The protocol was approved by the New York University Langone Medical Center Institutional Review Board (IRB) and the Bellevue Research Committee (BRC) of Bellevue Hospital Center.

### Airway epithelial cell collection and RNA processing

Through bronchoscopy, we collected peripheral airway epithelial cells by brushing the small airways in the lung contralateral to the suspicious nodule, i. e. the unaffected lung. In the controls without nodules, brushings were collected from the peripheral airways of the lingula or left lower lobe. The presence of Clara cells on the cytology brush confirmed sampling of the small peripheral airways (see S1 Fig in the online supplement). The cytology brush was spun down and the cell pellet was collected and stored at -80°C until RNA extraction.

### MicroRNA and mRNA microarray

We extracted total RNA using Qiagen miRNeasy Mini Kit (Valencia, CA). To identify target genes, global gene expression profiling was performed with the Affymetrix (Santa Clara, CA) GeneChip Human Genome U133 Plus 2.0 Array (HG-U 133 Plus 2.0). We used TaqMan Array Human MicroRNA A Cards v2.0 (Applied Biosystems, Foster City, CA) to profile miRNA on the same RNA samples. All microarray data have been submitted to the Gene Expression Omnibus (GEO) under accession number GSE54495 (mRNA) and GSE54541 (miRNA).

### Analysis of microarray mRNA and miRNA data

Affymetrix array data was analyzed by GeneSpring GX version 12.6.1 (Agilent Technologies, Inc., Santa Clara, US) using Linear Models for Microarray Data (LIMMA). MiRNA Taqman array data was normalized based on comparative CT representation using the global normalization method. DAVID 6.7 was used for functional enrichment analysis of mRNA data. Gene Set Enrichment Analysis (GSEA) was used to assess concordance between our data and *a priori* defined set of genes. DIANA-mirPath [20] was performed on miRNA TaqMan array data for Kyoto Encyclopedia of Genes and Genomes (KEGG) pathway analysis. Hierarchical heatmaps were generated with complete linkage clustering method and squared Euclidean distance measure. Ingenuity Pathway Analysis (IPA) was used to identify top biological functions and disease/disorders, and to generate pathways regulated from integrated mRNA-miRNA data (http://www.ingenuity.com). MiRNA predicted targets for integrated analysis were generated based on TargetScan 6.2 (www.targetscan.org) or miRanda (microRNA.org). (S2 Fig for overall study design). We used support vector machines (SVM) to develop multivariate molecular signatures of lung field of cancerization from differentially expressed miRNA and mRNA following the leave-one-out cross-validation protocol.

### Quantitative Real-time Reverse transcription PCR Analysis

We isolated total RNA using Qiagen miRNeasy Mini Kit (Valencia, CA) according to the company protocol, and performed quantitative real time-polymerase chain reaction on four mRNAs (ASCL1, AMOTL2, CLCN3, and MAP3k8) and four miRNAs (miR-486–3p, miR-483–5p, miR-374a, and miRNA-375).

### Statistical Methods and Graphs

We used GeneSpring and MATLAB (The MathWorks, Inc.) to calculate LIMMA—value, student *t*-test *p*-value, Benjamini-Hochberg False Discovery Rate (FDR) for adjustment of multiple comparisons, and fold change (FC) for each individual gene/miRNA probe. Correlation between phenotype and miRNA/mRNA was assessed using both correlation coefficient and partial correlation coefficient conditioned on age, sex, and smoking status. Differential expression comparison was made between early stage I and II adenocarcinoma vs. late stage III and IV adenocarcinoma. Pearson’s correlation was used to calculate correlation between RT-PCR and microarray platforms. Rank-order analysis was used to compare miRNA-mRNA correlation. Area under the curve was generated using Mann-Whitney U statistics theory.

(See S1 Methods for more detailed description)

## Results

### Demographic and Clinical Characteristics

Of the 30 subjects recruited for this study, 17 were diagnosed with lung adenocarcinoma after initial bronchoscopy and 13 were determined to be smoker controls, free of lung cancer. The clinical characteristics of these subjects are shown in Table 1. Most of the study subjects were male, Caucasian and had a significant smoking history (>30 pack years), and most cancers were stage III-IV lung adenocarcinoma.

**Table 1:** Demographic & clinical characteristics.

	Adeno Ca (n = 17)	Control (n = 13)	p-value†
**Age, yr (SD)**	64 (7.4)	62 (6.9)	0.41
**Male Sex (%)**	13 (76)	9 (69)	0.67
**Race**			0.46
**White**	14	9	
**African American**	1	3	
**Hispanic**	1	1	
**other**	1	0	
**Smoking status**			0.69
**Past**	11	10	
**Current**	6	3	
**Pack-years (SD)**	38 (19)^‡^	43 (23)	0.61
**Cancer Stage**			
**I**	3	na	
**II**	1	na	
**III**	4	na	
**IV**	9	na	

*Definition of abbreviations*: *Adeno Ca = adenocarcinoma*

Mean and standard deviation (SD) were calculated.

^†^p-values calculated using Student's t-test or Fisher exact test.

^‡^Missing pack-years for 1 subject

### Gene Expression in the Peripheral Airways Contralateral to the Tumor

**Fig 1:**
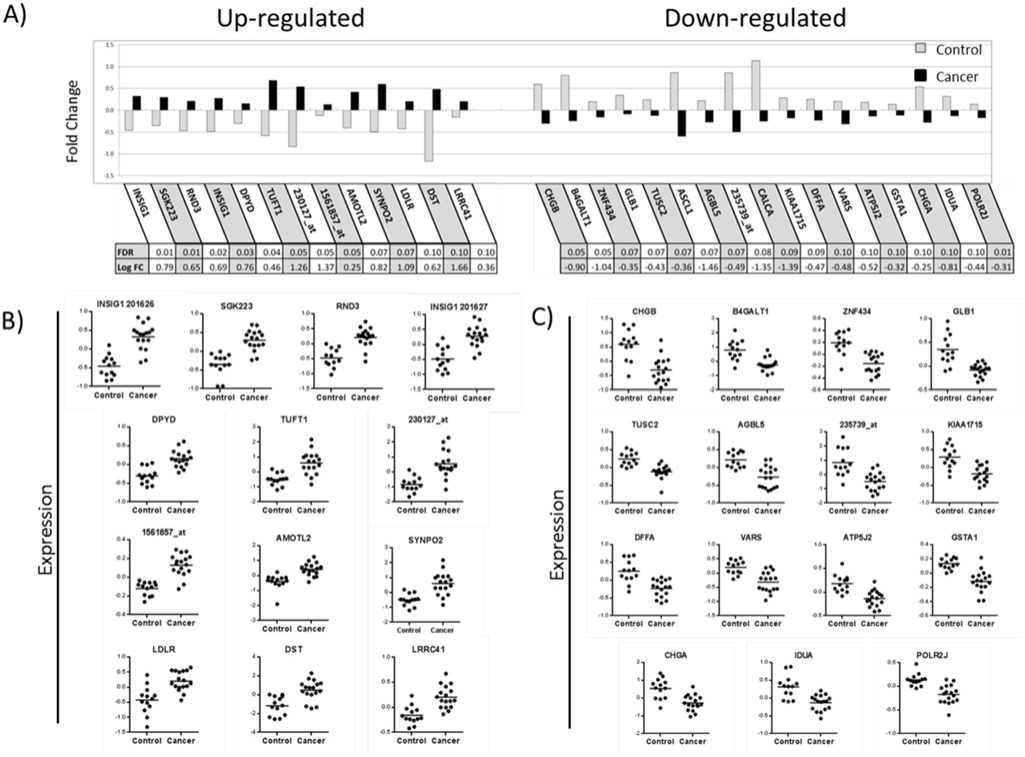
Top 30 Gene expressions in the peripheral airway epithelial cells. **A)** Using Affymetrix Gene Chip HG U133 Plus 2.0, Top 30 differentiated mRNAs in peripheral airway field of cancerization of cancer patients **(black bar)** vs. peripheral airways of smoker controls **(gray bar)** are shown. 13 mRNAs were strongly up-regulated and 17 mRNAs were strongly down-regulated in peripheral airway field of cancerization. Gene expression fold change is shown as the mean expression relative to a reference value. Table below shows FDR value base on Benjamini-Hochberg multiple testing and log2 fold change value of cancer patient relative to smoker control. **B)** Individual dot plot of cancer patients (n = 17) compared to smoker controls (n = 13) with **up-regulated** mRNAs. **C)** Individual dot plot with **down-regulated** mRNAs.

To characterize differences in gene expression found in the field of cancerization in lung cancer patients compared to smoker controls, we collected peripheral airway epithelial cells from airways distant from the tumor, *i*.*e*. in the contralateral lung, which we term the contralateral peripheral airway. RNA samples were extracted and run on an Affymetrix HG-U 133 Plus 2.0 chip. After adjusting for age, sex, and smoking status, we used FDR correction of 0.10 as the threshold. 64 mRNAs in the contralateral peripheral airway epithelial cells were found to be differentially expressed between lung cancer patients and smoker controls (S1 Table); the top 30 mRNAs are shown in Fig. 1A. Of these 64 mRNAs, 38 mRNAs were down-regulated in lung cancer patients, of which the top 5 mRNAs were CHGB, B4GALT1, ZNF434, GLB1, and ASCL1 (*p*≤0.0001). Of the 26 mRNAs that were up-regulated, INSIG1, SGK223, RND3, TUFT1, DPYD, and AMOTL2 were the most significant (*p*≤0.0001). Fig. 1B-C shows individual dot plots for each of the top 30 mRNAs identified as significantly different between cases and controls. These findings suggest possible differences in underlying biological processes.

To further evaluate the biological processes occurring in the peripheral airway field of cancerization, we performed DAVID Bioinformatic functional enrichment analysis from the above gene list. Functional Annotation analysis (S2 Table) with a “medium” classification stringency generated a list of biological processes of which the top terms included apoptosis (fold enrichment = 3.9, p-value = 0.01), programmed cell death (fold enrichment = 3.9, p-value = 0.01), disaccharide metabolic process (fold enrichment = 133.1, p-value = 0.01), cell death (fold enrichment = 3.3, p-value = 0.02), and cell proliferation (fold enrichment = 3.9, p-value = 0.04). Apoptosis, programmed cell death, and cell death genes included TNS4, BAD, AHR, B4GALT1, and PLG. Cell Proliferation genes included CALCA, TUSC2, ASCL1, INSIG1, and BAD. These differentially expressed biological processes in the contralateral peripheral airway, many of which are important in tumorigenesis, suggest that tissue far from the tumor may also have abnormal molecular expressions. We performed GSEA using publicly available datasets. We identified concordant relationship with 10 gene sets (FDR<0.1) in the C4 computational gene sets, cancer gene neighborhoods collection by Broad Institute Molecular Signatures Database (MSigDB). These gene sets are involved in apoptosis (MORF_DAP, MORF_CSNK2B, MORF_PHB), DNA repair (MORF_DDB1), and oncogenes MYC (MORF_NME2) and RAS (MORF_RAN). (S3 Fig, S3 Table). We also identified 14 gene sets (FDR<0.1) in the C4 computation gene sets, cancer modules collection by MSigDB (more details in S4 Table). Top 5 gene sets are shown in S4 Fig. Both DAVID and GSEA identified similar biological processes in the peripheral airway field of cancerization, many of which relate to lung cancer pathogenesis.

**Fig 2:**
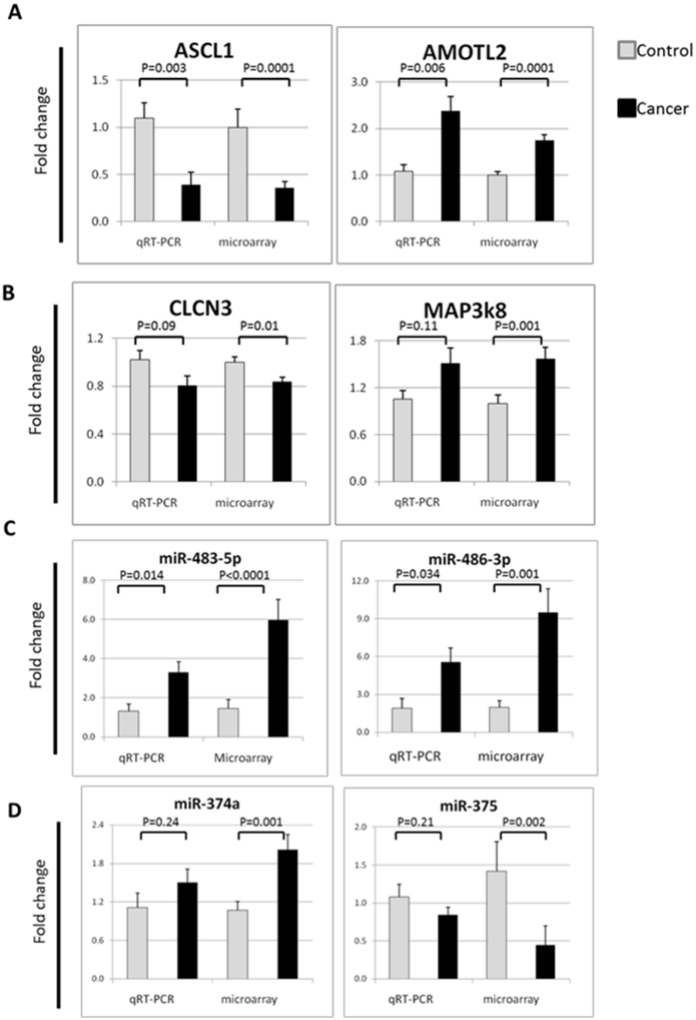
Real time RT-PCR validation. **A)** Two highly significant differentially expressed genes (ASCL1 and AMOTL2) detected by microarray in the peripheral airway epithelial cells of cancer patients compare to smokers controls, were confirmed with RT-PCR, producing similar p-value and fold change. **B)** Two randomly selected genes (CLCN3 and MAP3k8) were confirmed with RT-PCR to produce fold change in same direction and with similar magnitude to that seen with microarray. Gene expressions were expressed as means and ± standard errors of means. **C-D)** Three up-regulated miRNA and a fourth down-regulated miRNA were select for RT-PCR to confirmation miRNA expression. miR-486–3p, miR-483–5p, and miR-374a were up-regulated and miR-375 was down-regulated in the peripheral airway field of cancerization in lung cancer patients compared to smoker controls; fold change between cancer patients and smoker controls were in the similar direction. MiRNA expressions were expressed as means and ± standard errors of means.

We next used quantitative RT-PCR to confirm our Affymetrix microarray results. First, we validated two of the most significantly altered genes, ASCL1 and AMOTL2, in a subset of 20 subjects (13 lung cancer patients and 7 controls). The different gene expression detection methods produced similar *p*-values and fold changes (Fig. 2A). ASCL1 was significantly decreased in cancer compared to control by 0.32-fold, p = 0.003 (RT-PCR) vs. 0.36-fold *p* = 0.0001 (Affymetrix microarray). AMOTL2 was significantly increased in cancer compared to control by 1.2-fold, *p* = 0.006 (RT-PCR) vs. 1.7-fold, *p* = 0.0001 (Affymetrix microarray). Similarly, we used RT-PCR to evaluate expression of two other randomly selected genes, CLCN3 and MAP3k8, and found fold change in the same magnitude and direction using the two methods (Fig. 2B). CLCN3 was decreased in cancer compared to control by 0.80-fold, *p* = 0.09 (RT-PCR) vs. 0.84-fold, *p* = 0.01 (microarray). MAP3k8 was increased in cancer compared to control by 1.51-fold, *p* = 0.11 (RT-PCR) vs. 1.57-fold, *p* = 0.001 (microarray). Correlation plots between RT-PCR data and microarray data for individual subjects are shown in S5 Fig.

It is interesting to note that when we compared our samples based on tumor stage, analysis of stage I/II (early) vs. stage III/IV (late) did not produce statistically significant mRNAs (FDR<0.1); suggesting that pathogenesis of field of cancerization may differ from that of tumor metastasis. It is possible that this study may not be powered to analyze gene expression differences between tumor stages; alternatively, it is possible that no differences were detected because there was no direct metastasis to the contralateral lung in our subjects.

### MicroRNA expression in the Peripheral Airways Contralateral to the Tumor

**Fig 3:**
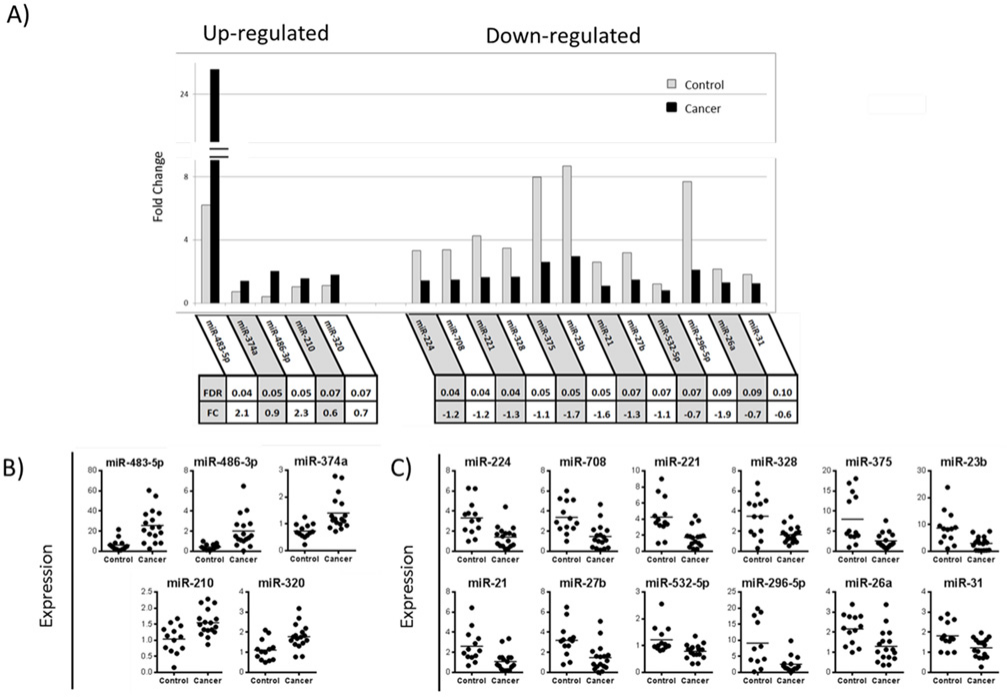
MicroRNA expression in the peripheral airway epithelial cells. **A)** Using TaqMan miRNA Array, 5 miRNAs were highly (FDR <0.1) up-regulated in peripheral airway field of cancerization of cancer patients **(black bar)** vs. peripheral airways of smoker controls **(gray bar).** 12 miRNAs (FDR<0.1) were strongly down-regulated. MiRNA fold change is shown as the mean expression relative to a reference value. Table below shows FDR value base on Benjamini-Hochberg multiple testing and log2 fold change value of cancer patient relative to smoker control. **B)** Individual dot plot of cancer patients (n = 17) compared to peripheral airways of smoker controls (n = 13) with **up-regulated** miRNAs. **C)** Individual dot plot with **down-regulated** miRNAs.

We used the same RNA samples extracted from the contralateral peripheral airway epithelial cells of lung cancer patients and smoker controls to profile miRNA expression using TaqMan Array Human MicroRNA A Cards v2.0. Adjusting for age, sex, and smoking status, and using FDR < 0.1 filter, 17 miRNAs were found to be differentially expressed (Fig. 3A). Of these 17 miRNAs (S5 Table), 12 miRNAs were down-regulated in lung cancer patients; miR-224, miR-708, miR-221, and miR-328 were the most significant. Of the 5 miRNAs that were up-regulated in cases with lung cancer, miR-483–5p, miR-374a, and miR-486–3p were the most significant. Fig. 3B-C shows individual dot plots for each of the 17 miRNAs. Differences in miRNA expression between cancer patients and smoker controls suggest that modulation of miRNAs may activate biological pathways relating to tumorigensis.

To explore the biological pathways that might be regulated by these miRNAs, we performed a KEGG pathway classification analysis using the DIANA-mirPath [20] with microT-CDS prediction. MicroT-CDS prediction identified the top five most significant KEGG pathways: ErbB signaling pathway (p = 2.3x10^-26^, 14 miRNAs), prostate cancer (p = 2.3x10–26, 14 miRNAs), TGF-beta signaling pathway (p = 2.1x10^-25^, 13 miRNAs), mitogen-activated protein kinase (MAPK) signaling pathways (p = 6.2x10^-49^, 24 miRNAs), and Pathways in cancer (p = 5.1x10^-21^, 16 miRNAs) (S6 Fig). Interestingly most KEGG pathways are due to four miRNAs: miR-374a, miR-26a, miR-27b, and miR-320a. Modulation of cancer related pathways support the presence of field of cancerization at the miRNA level in the contralateral peripheral airway epithelial cells.

To validate our microarray findings we randomly selected four miRNA to quantify by quantitative RT-PCR and compared with quantification of miRNA by TaqMan array in a subset of 19 subjects (12 lung cancer patients and 7 controls). The up-regulated miRNAs included: miR-486–3p, with 2.9 fold change (*p* = 0.034) by RT-PCR vs. 4.8 fold change (*p* = 0.001) by TaqMan array; miR-483–5p, with 2.5 fold change (*p* = 0.014) by RT-PCR vs. 4.1 fold change (*p*<0.0001) by TaqMan array; and miR-374a, with 1.3 fold change (*p* = 0.24) by RT-PCR vs. 1.9 fold change (*p* = 0.001) by TaqMan array (Fig. 2C-D). MiRNA-375 was down-regulated, with 0.8 fold change (*p* = 0.21) by RT-PCR vs. 0.3 fold change (*p* = 0.002) by TaqMan array. Correlation plots between RT-PCR data and TaqMan array data for individual subjects are shown in S7 Fig. Importantly, differences in miRNA expression were in the same direction for both methods. However, the magnitude of the difference between cases and controls was larger for the TaqMan array data.

### Integrated mRNA and miRNA analysis identified the ERK/MAPK Pathway

**Fig 4:**
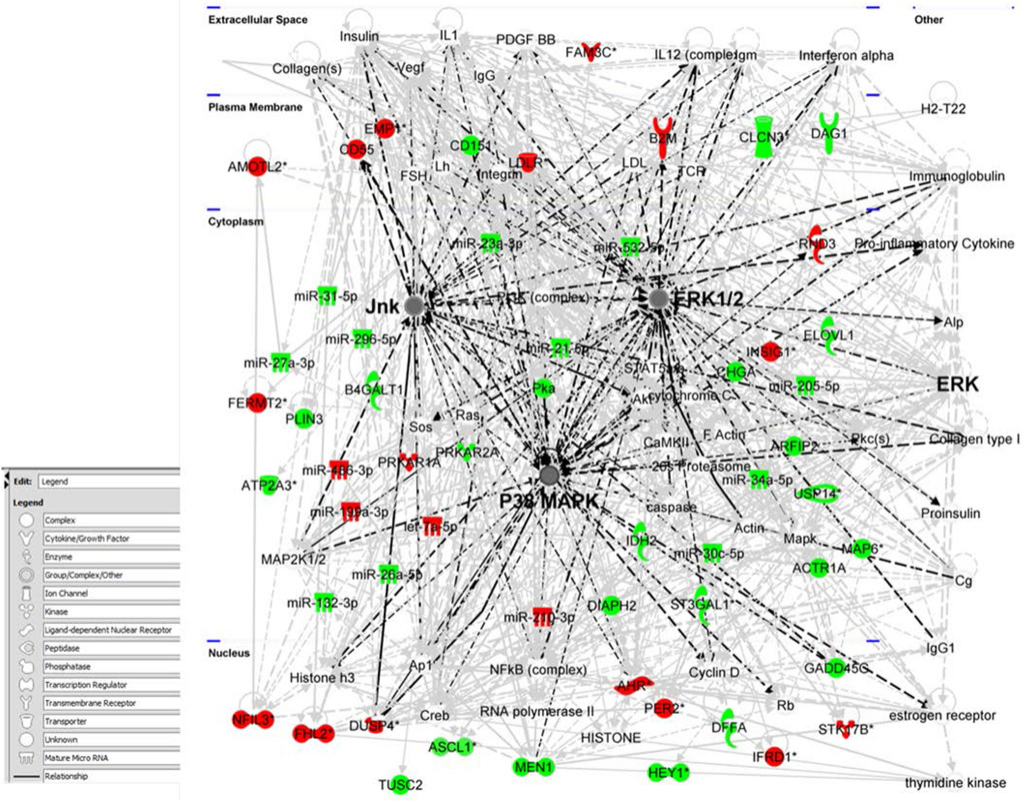
ERK/MAPK Signaling Pathway. IPA was used to generate a network of molecules from mRNA and miRNA that were significantly different in peripheral airway field of cancerization in lung cancer patients compared to smoker controls. This composite network merge three top networks based on “network score,” which converges on the ERK/MAPk pathway: specifically merging on Extracellular signal-related kinases 1 and 2 (ERK1/2), c-Jun N-terminal kinases (JNKs), and p38 kinases subfamily. **Red** is up-regulated and **Green** is down-regulated.

To further explore the pathways associated with the identified changes in uninvolved peripheral airway epithelial cells from cancer patients vs. smoker controls, we integrated differentially expressed miRNA data with the negatively correlated predicted mRNA targets from these 30 patients for analysis. Assuming larger statistical power by integrating miRNA and mRNA data, we selected negatively correlated miRNAs and mRNAs with FDR<0.15. A total of 24 miRNAs and 67 mRNAs probes were selected after filter, producing 102 miRNA-mRNA pairings (S6 Table). Using IPA program analysis, we identified the top biological functions of these miRNA-mRNA pairings as being involved in cellular development (*p* = 7.8x10^-8^, 38 molecules), cellular growth and proliferation (*p* = 2.5x10^-7^, 43 molecules), cell death and survival (*p* = 1.1x10^-6^, 44 molecules), cellular movement (*p* = 3.1x10^-5^, 31 molecules), and cell cycle (*p* = 2.6x10^-5^, 19 molecules). IPA network generation identified that the majority of these mRNA and miRNAs (54 molecules) were networked to the extracellular signal-regulated kinase/mitogen-activated protein kinase (ERK/MAPK) pathway (Fig. 4), which represents a composite of merging 3 top interaction networks based on “network score.” This higher-order analysis was able to identify a previously known oncogenic signaling pathway, MAPK, in the peripheral airway field of cancerization, suggesting that interaction between mRNA and miRNA may be important in field of cancerization.

### miRNA and mRNA expression correlation identifies miR-374a and ASCL1 interaction

**Fig 5:**
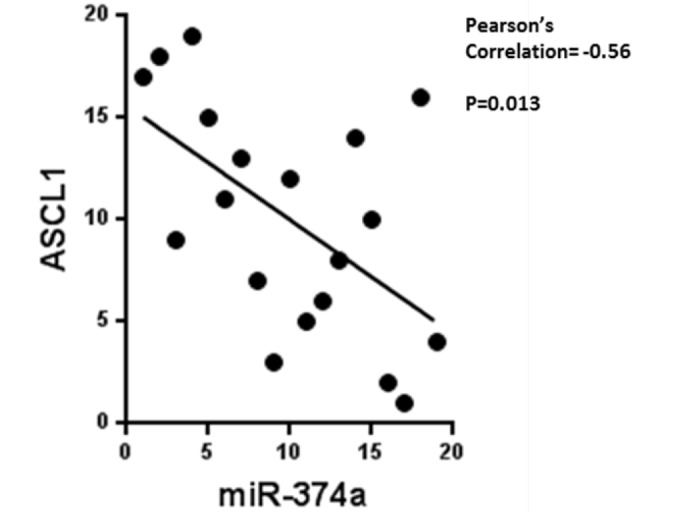
Correlation between miR-374a and ASCL1. Quantitative RT-PCR for miR-374a and ASCL1 were performed in each individual’s peripheral airway epithelial cells (n = 17 cancer patient and n = 13 smoker controls) to confirm miRNA-mRNA negative correlation in the array. We confirm that ASCL1 expression was negatively correlated with miRNA-374a expression in peripheral airway epithelial cells (Pearson’s = -0.56, p = 0.013).

To further characterize miRNA-mRNA interaction in the peripheral airway field of cancerization, we explored specific miRNA-mRNA pairings. Vosa and colleagues identified miR-374a as a prognostic biomarker in early stage non-small cell lung cancer [21]. We also identified miR-374a as a differentially expressed miRNA in the peripheral airway field of cancerization; its expression was increased in lung cancer patients compared to smoker controls (Fig. 3). Using a publicly available miRNA target prediction program, miRanda, we searched for possible targets of miR-374. One of the predicted targets, ASCL1, a gene member of the basic helix-loop-helix family transcription factors, was also identified in our study as differentially expressed. Quantitative RT-PCR confirmed our microarray results (Pearson’s correlation = 0.47, p = 0.04) (S7 Fig). When we correlated the miR-374a and ASCL1 quantitative RT-PCR expressions in each individual, we found a significant negative correlation (p = 0.013), as expected with miRNA-mRNA pairings (Fig. 5.) While there is substantial literature describing the function of ASCL1, little is known about miR-374a. We know that ASCL1 is important in pulmonary neuroendocrine cell development, lung injury repair, airway dysplasia, and neuroendocrine differentiated lung cancer pathogenesis [22–24]. There is some evidence that low miR-374a expression level in non-small cell lung cancer is associated with poor survival, suggesting it may have tumor suppressive effect. We hypothesize that miR-374a is up regulated in the field of cancerization, and in turn down regulates ASCL1 transcription factor, which may represent a reaction of epithelial cells to the presence of tumor in the lung; possibly counteracting and/or preventing tumor metastasis. It would be important to explore further the mechanism by which this interaction affects tumor invasion.

### Molecular Signatures in the Lung Field of Cancerization

To explore whether differentially expressed mRNAs and miRNAs in the contralateral peripheral airway epithelial cells could be used to differentiate our cancer patients from cancer-free smokers, we developed molecular signatures from mRNA and miRNA data using support vector machines (SVM) following the leave-one-out cross-validation (LOOCV) protocol. When SVMs were applied to differentially expressed mRNAs (FDR<0.15 and |FC|>1.5, as determined by LOOCV), a receiver operating characteristic (ROC) curve with area under the curve (AUC) of 0.92, 95% CI 0.82–1.00, p<0.0001 was generated (S8A Fig). On average, 30 mRNAs were included to generate these molecular signatures. Using miRNAs data, SVM molecular signatures were based on 24 miRNAs, on average, and generated a ROC curve with AUC = 0.94, 95% CI 0.86–1.00, p<0.0001 (S8B Fig). The top mRNAs and miRNAs selected are listed in S8C Fig and S8D Fig. These results, although only performed in a training set, suggest that both mRNAs and miRNAs in the contralateral peripheral airway epithelial cells differentiate lung cancer patients from smoker controls in our cohort, and could be used as a biomarker for diagnosis. Therefore, performance evaluation of these molecular signatures in a validation set is needed.

## Discussion

In this study we showed that peripheral airway epithelial cells distant from the lung tumors have many differentially expressed mRNAs and miRNAs commonly associated with tumorigenesis when compared with peripheral airways from smoker controls. The transcriptomic profile of these epithelial cells showed cellular functions relating to cell adhesion (DST, RND3) [25,26], apoptosis (NR4A2, STK17B, miR-21) [27–29], airway dysplasia (ASCL1) [23], tumor suppression (MAP3k8) [30], angiogenesis (AMOTL2) [31], and signaling pathways, such as MAPK signaling (miR-483–5p, LDLR) [32,33], and PI3k-Akt (IRS2, miR-26a) [34,35]. We also identified several miRNAs in the peripheral airways that have been implicated in carcinogenesis. For example, miR-483–5p, previously identified as being abnormally expressed in hepatocellular carcinoma and rectal cancer[36,37], exerts its effect by directly targeting the ERK1 pathway [33], and was recently shown to be important in lung cancer cell proliferation and senescence[38]. We identified miR-483–5p as the top differentially expressed miRNA in our miRNA array and confirmed its expression by quantitative RT-PCR (Figs. 2C and 3), also suggesting that miR-483–5p may play a role in the peripheral airway field of cancerization. In addition, we observed that 71% (12/17) of the differentially expressed miRNAs were down-regulated. This result reflects current evidence across multiple different cancer types that there is a global down-regulation of miRNAs in cancer [39,40]. Mascaux and colleagues examined bronchial biopsies from a wide range of patients including never smokers, smokers with normal bronchial epithelium, and patients with bronchial dysplasia, carcinoma *in situ*, and invasive squamous cell carcinomas, and found a linear reduction in miR-32 and miR-34c expression correlating with progression from normal bronchial epithelium to squamous cell carcinoma [41]. This is an interesting finding suggesting that global down-regulation of miRNAs may play a role in tumorigenesis. If true, further studies regarding miRNA expression in the field of cancerization may provide potential targets for chemopreventive strategies for lung cancer [42].

By integrating miRNA and mRNA data in the field of cancerization, we showed for the first time that the MAPK signaling pathway is deregulated in the contralateral peripheral airway epithelial cells. MAPK signaling is comprised of three kinases: ERK kinases 1 and 2 (ERK1/2), c-Jun N-terminal kinase (JNKs), and p38 kinase. ERK1/2 MAPK signaling is highly responsive to growth factors and cytokines, resulting in cell proliferation, cell division, and cell differentiation [43,44]. Activation of ERK1/2 has been shown to correlated with advanced and aggressive NSCLC [45]. Dubinett et al. demonstrated that the ERK1/2 pathway appears to be a potential intermediary of the Snail-mediated up-regulation of SPARC, which contributes to lung tumor invasion and migration [46]. In contrast, JNK and p38 kinases respond to ultraviolet radiation, oxidative stress, and tumor necrosis factor, leading to apoptosis, inflammation, and cell cycle arrest. Khatlani et al. showed that JNK is activated in NSCLC cancer and promotes oncogenesis in bronchial epithelial cells [47]. We also previously reported that activated p38 kinases are up-regulated in NSCLC tissue by Western blotting [48]. Our microarray identified two molecules that were shown to affect MAPK signaling: DUSP4 and IRS2, both have previously been shown to modulate ERK1/2 activation [49,50]. This example of integrated miRNA-mRNA analysis highlights the utility of combining integrated data for functional genomics to identify novel molecules and pathways in field of cancerization. We also identified a possible mRNA/miRNA pairing in ASCL1 and miR374a. Further research is needed to better understand the mechanism of this interaction, which we hypothesized, may be involved in suppressing tumor invasion, based on known functional properties of these two molecules. Our findings, although not confirmed through functional experiments, are supported by our quantitative RT-PCR data on peripheral airway epithelial cells and strengthen the idea that molecular differences seen in the contralateral peripheral airway epithelial cells between cancer patients and smoker controls may be involved in pathogenic pathways for lung cancer. This concept has not yet been described in areas far distant from the lung tumor.

We also evaluated our results in the context of previously published studies of airway gene profiling in lung cancer. In Kadara’s study which profiled field of cancerization in non-small cell lung cancer subjects, the investigator showed that the ERK/PI3K pathways were up-regulated in epithelial cells of adjacent airways compared to the contralateral airways [51,52]. We compared the gene expression of our 64 mRNAs to the transcriptomic architecture from Kadara’s airway epithelial brushing “farthest away” from the tumor (GSE44077), and identified 38 mRNAs which were differentially expressed in both data sets (FDR <0.1). Of these, 61% (23/38) were up or down regulated in the same direction (S7 Table). Interestingly, we found only 36% (16/45) of mRNAs were similar in up/down regulation when we compared to Kadara’s airway epithelial brushing “closest” to the tumor (FDR <0.1). Our data supplement Kadara’s finding of a gradient field of cancerization effect relating to the proximity of the airway to the tumor, by suggesting that this gradient effect may extend to the contralateral peripheral airway. However, it is important to note the differences in study design between the two studies: while our study was designed to compare differences between cancer patients and smoker controls, Kadara’s comparisons were made within samples from cancer patients only. Therefore we can only infer possible correlation when comparing these two data sets. In a separate analysis, we also compared gene expression of proximal large airway epithelial cells (GSE4115) [17] to our data set and were able to identify some similar gene expressions (S8 Table). Spira et al. demonstrated PI3k pathway activation in the proximal airway during and prior to lung cancer development [53]. Our analysis extends current knowledge regarding field of cancerization by identifying a list of commonly differentiated mRNAs expressed in the proximal large airway, adjacent airways, and contralateral peripheral airways; providing a spatial gene expression map to help identify potentially important mechanisms in lung cancer pathogenesis.

Of the 64 mRNAs and 17 miRNAs significantly expressed in the contralateral peripheral airway, 10 mRNAs/miRNAs have similar up/down expression as that of lung adenocarcinoma tumors or lung adenocarcinoma cell lines; AHR (up), DPYD (up), ERV3–1 (up), LDLR (up), TUSC2 (down) miR-224 (down), miR-27b (down), miR-26a (down), miR-483–5p (up), and miR-210 (up) [54–61]. This suggests that some oncogenic expressions can be detected in areas some distance away from the lung tumor, in the contralateral peripheral airway epithelial cells. While Kadara el al. was able to show a spatial gradient effect of cancerization, it is reasonable to think that our data supplement their findings by suggesting that although field of cancerization can be detected some distance away, it is more likely that it follows a gradient in which a decreasing effect with increasing distance from the tumor is observed. We hypothesize that while cigarette smoke causes a whole field of injury in the lung, only those at-risk for developing lung cancer have an increase of oncogenic cell-lineage in many of the peripheral airway epithelial cells. However, a focal area with the highest oncogenic expression might eventually arise, transforming into a self-replicating colony and eventually becoming cancerous; this may be a possible explanation for the gradient effect in the lung.

However, it is important to consider other hypotheses as to the importance of field of cancerization and its role in tumorigenesis. For example, it is interesting to note that there are mRNAs and miRNAs with opposite up/down expression compared to lung adenocarcinoma tumors / cell lines: AKIP1 (down in field of cancerization vs. up in lung adenocarcinoma), ASCL1 (down vs. up), miR-708 (down vs. up), miR-21 (down vs. up), and miR-31(down vs. up) [59,62–65]. It is reasonable to consider that while some of the pro-oncogenic molecules are expressed in the field of cancerization, some anti-oncogenic molecules may actually reflect a counter-regulatory mechanism in the contralateral lobe in response to the presence of tumor in the lung; either to prevent metastasis of the lung tumor or to counteract elevated carcinogenic pathways. Alternatively, this observed opposite expression may reflect progenitor cell “fatigue” or “burn-out.” [66]. For example, Achaete-Scute homologue-1 (ASCL1), a pro-neural transcription factor, is important in pulmonary neuroendocrine cell development [22], injury repair [24], and highly expressed in neuroendocrine differentiated lung cancer functioning as a regulator of RET oncogene [65]. Following constant insult from cigarette smoke damage, it is possible that adult ASCL1 defined cells, may suffer from “fatigue,” possibly resulting in reduced capacity of progenitor cells to successfully differentiate into normal epithelial cells. Clearly, more experiments are needed to further explain this complex system.

Our study was limited by the moderate sample size which may have limited the power to detect additional significant differentially expressed mRNA, miRNA, and/or miRNA-mRNA pairings between cancer patients and smoker controls. Also, additional sampling of multiple lung regions, including the tumor itself, tumor-adjacent, ipsilateral, and contralateral airways could more definitively illustrate the spatial effect of field of cancerization. Nevertheless, field of cancerization has the potential be a source of biomarker discovery. While we believe that our method of sampling of the peripheral airway epithelial is a minimally invasive procedure, we acknowledge that currently this method of biomarker discovery is not clinically optimal or practical as it cannot be routinely performed. Currently substantial lung cancer biomarker research has been conducted in the large airway bronchial cells [17], and future work should also focus on oral and nasal epithelial cells as potential areas affected by lung cancer field of cancerization, given the fact that these regions can be sampled easily.

In summary, we profiled the global mRNA and miRNA expression of the peripheral airway epithelium contralateral to the tumor in lung cancer patients and compared it to smoker controls. We showed that in these presumably histologically normal peripheral airway epithelial cells, our method identified changes in the molecular expression of genes relating to cellular processes important in tumorigenesis, such as apoptosis, cell proliferation, and DNA repair. We also demonstrated that using our technique, we were able to detect modulations in known candidates important to tumorigenesis—MAPK signal pathway in the contralateral lung of the tumor. Future studies on mechanisms are needed in the field of cancerization to proof this concept and to provide insights on changes in different cell types, such as bronchoalveolar stem cells which may represent the early steps by which epithelial damage leads to dysplasia and ultimately tumor initiation. The concept of field of cancerization will continue to evolve as more molecular information emerges. We believe that better understanding of field of cancerization will help improve the poor survival rate in lung cancer patients.

## Supporting Information

S1 Fig Peripheral Airway Epithelial Brushing. (DOCX)

S2 Fig Study Design. (DOCX)

S3 Fig Gene Set Enrichment Analysis (GSEA) enrichment plot: cancer gene neighborhoods. (DOCX)

S4 Fig Gene Set Enrichment Analysis (GSEA) enrichment plot: cancer module. (DOCX)

S5 Fig Correlation plot of RT-PCR vs. microarray platform. (DOCX)

S6 Fig Hierarchical heatmap of significant KEGG pathway based on differentiatially expressed miRNA in the peripheral airway field of cancerization with *micro-T-CDS prediction*. (DOCX)

S7 Fig Correlation plot of RT-PCR vs. TaqMan array platform. (DOCX)

S8 Fig Molecular Signatures in Peripheral airway Field of Cancerization. (DOCX)

S1 Table Differentially expressed genes with Affymetrix Gene Chip HG U133 Plus 2.0, FDR <0.1. (DOCX)

S2 Table DAVID functional enrichment analysis. (DOCX)

S3 Table Gene Set Enrichment Analysis (GSEA) with MSigDB C4 cancer gene neighborhoods. (DOCX)

S4 Table Gene Set Enrichment Analysis (GSEA) with MSigDB C4 cancer gene modules. (DOCX)

S5 Table Top differentially expressed microRNAs with TaqMan miRNA Array. (DOCX)

S6 Table Integrative negatively correlated mRNA-miRNA pairings base on TargetScan. (DOCX)

S7 Table Transcriptomic profile comparison to Kadara et al. (DOCX)

S8 Table Gene expressions comparison between peripheral airway epithelial cells and large airway epithelial cells. (DOCX)

S1 Methods Supplemental methods. (DOCX)
