# Supplementary material for: Molecular Characterization of the Peripheral Airway Field of Cancerization in Lung Adenocarcinoma
Source: PLoS One. 2015 Feb 23;10(2):e0118132. doi: 10.1371/journal.pone.0118132 (PMC4338284; doi:10.1371/journal.pone.0118132)

**S1 Figure. Peripheral Airway Epithelial Brushing.** Cytology brush was advanced distally to the small peripheral airway beyond the fourth-sixth order bronchial branching of the lung contra-lateral to the suspicious nodule. Low power (100x) view of small peripheral airway epithelial cells **(black arrow head)** and Clara cells **(white arrows)**.


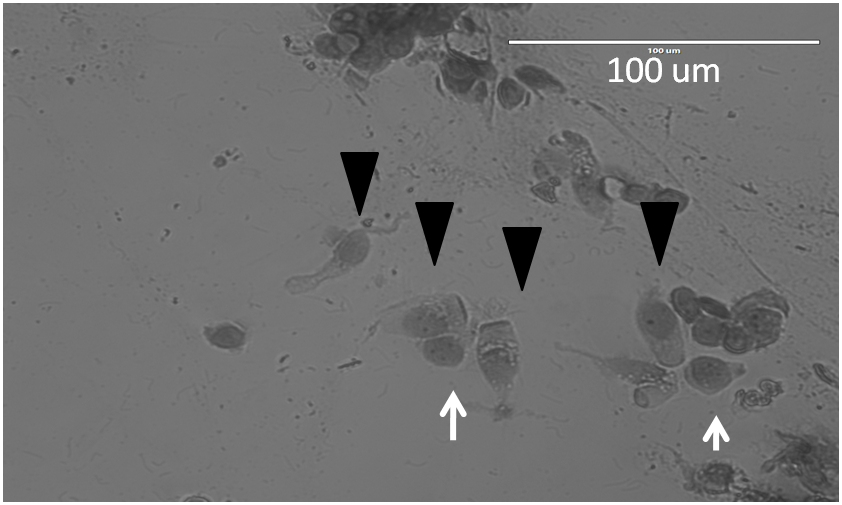

Supplement: S1 Fig — (DOCX) [file pone.0118132.s001.docx]
