# Supplementary material for: Molecular Characterization of the Peripheral Airway Field of Cancerization in Lung Adenocarcinoma
Source: PLoS One. 2015 Feb 23;10(2):e0118132. doi: 10.1371/journal.pone.0118132 (PMC4338284; doi:10.1371/journal.pone.0118132)

**S2 Figure. Study Design.** Transcriptomics of peripheral airway epithelial cells from the non-tumor lung of cancer patients were analyzed and compared with peripheral airway epithelial cells of control smokers. Affymetrix microarray and TaqMan array were performed on all samples (n=30). DAVID and GSEA were used for functional enrichment analysis on mRNA Affymetrix data. DIANA-mirPath was used for KEGG pathway analysis on miRNA TaqMan data. Support vector machines (SVM) used to develop multivariate molecular signatures. Leave-one-out cross-validation (LOOCV) protocol was used to develop molecular signature and estimate their classification performance. Ingenuity Pathway Analysis was used to analyze oncogenic pathways on integrated mRNA-miRNA data.


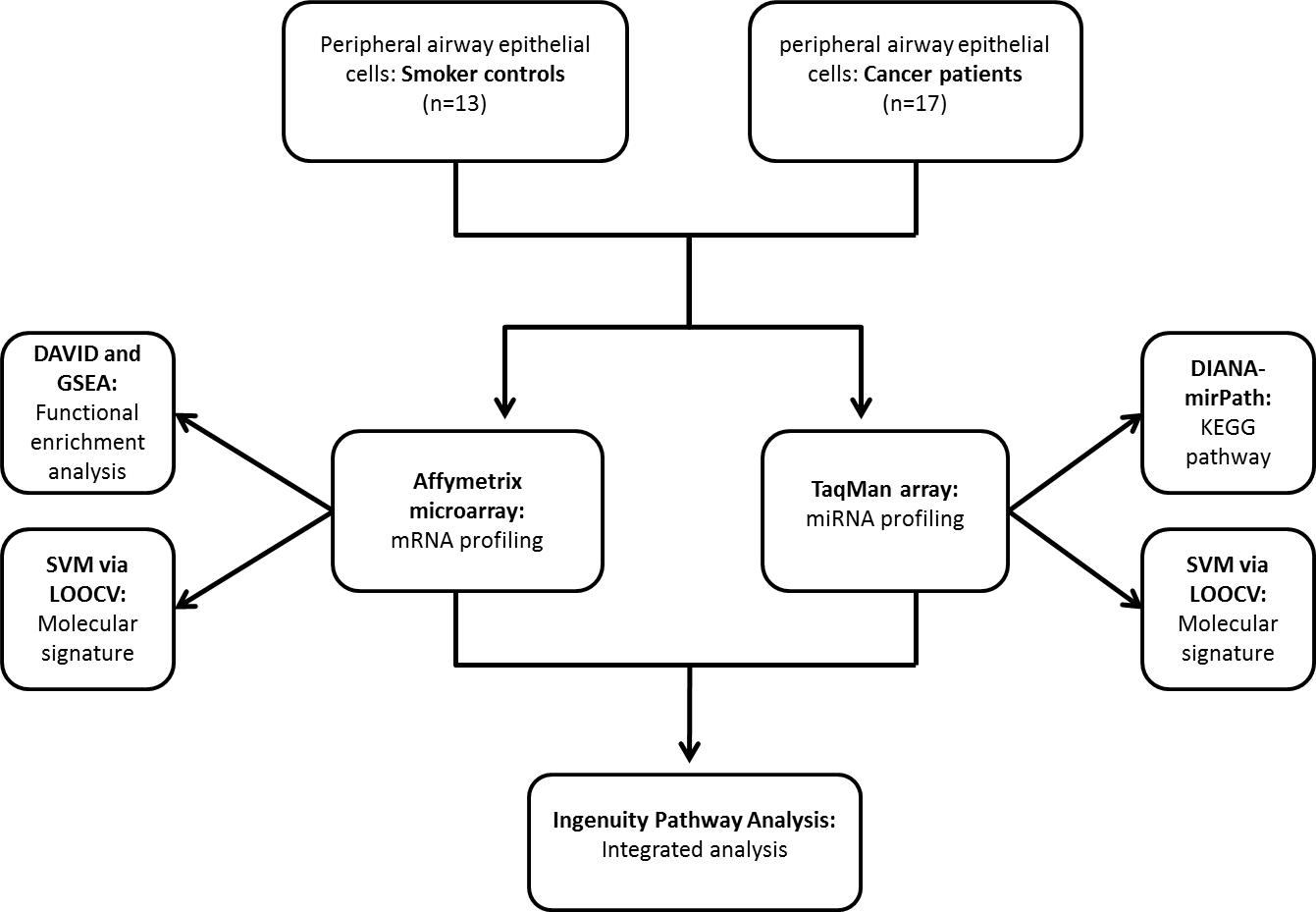

Supplement: S2 Fig — (DOCX) [file pone.0118132.s002.docx]
