# Supplementary material for: Molecular Characterization of the Peripheral Airway Field of Cancerization in Lung Adenocarcinoma
Source: PLoS One. 2015 Feb 23;10(2):e0118132. doi: 10.1371/journal.pone.0118132 (PMC4338284; doi:10.1371/journal.pone.0118132)
Supplement: S3 Fig — (DOCX) [file pone.0118132.s003.docx]

**S3 Figure. Gene Set Enrichment Analysis (GSEA**) **enrichment plot:** Top 5 gene sets from MSigDB C4 cancer gene neighborhoods (CGN) collection, which were concordant with the peripheral airway field of cancerization.

**
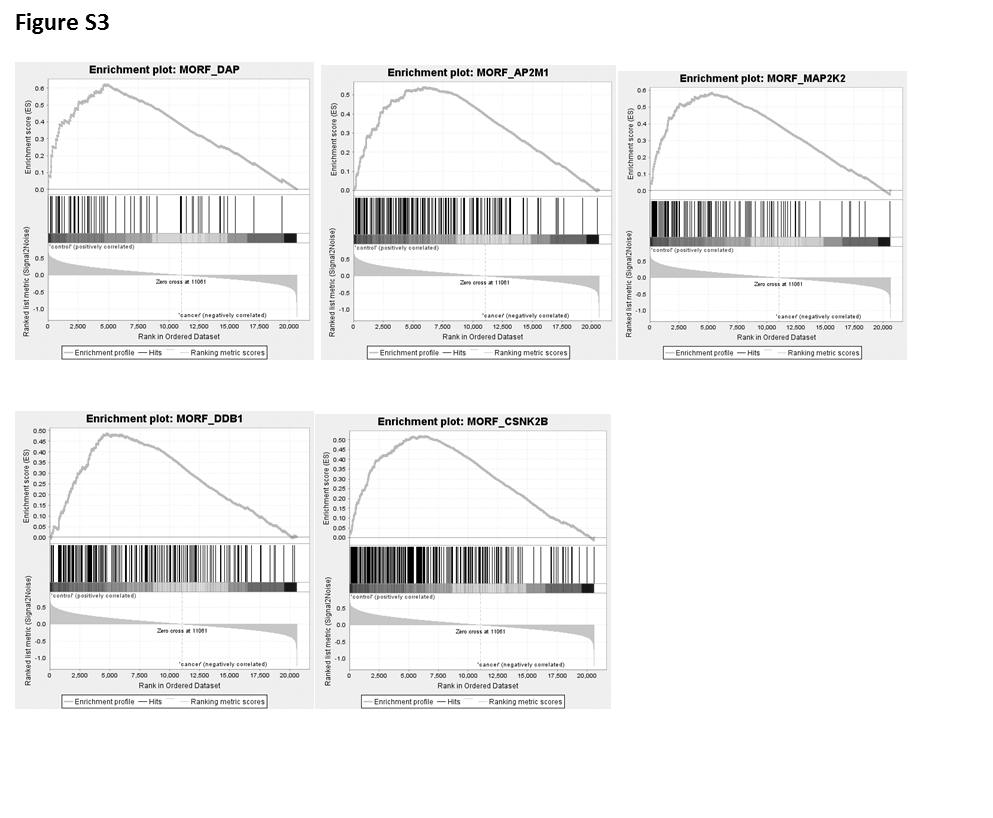
**
