# Supplementary material for: Molecular Characterization of the Peripheral Airway Field of Cancerization in Lung Adenocarcinoma
Source: PLoS One. 2015 Feb 23;10(2):e0118132. doi: 10.1371/journal.pone.0118132 (PMC4338284; doi:10.1371/journal.pone.0118132)
Supplement: S5 Fig — (DOCX) [file pone.0118132.s005.docx]

**S5 Figure. Correlation plot of RT-PCR vs. microarray platform.** Plots of individual subjects show good correlation between RT-PCR data and microarray data for ASCL1, AMOTL2, CLCN3, and MAP3k8. Statistical calculation with Pearson’s correlation.

**
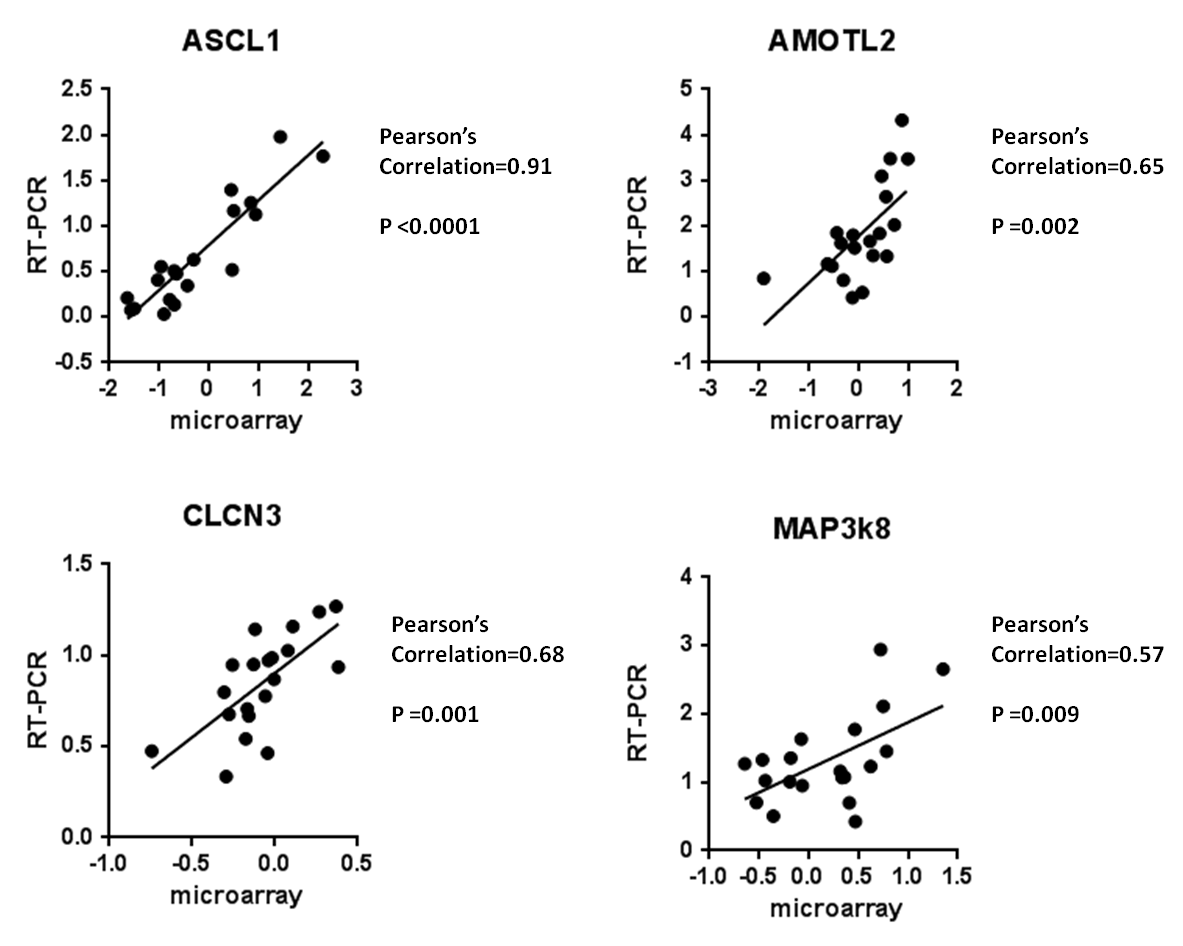
**
