# Supplementary material for: Molecular Characterization of the Peripheral Airway Field of Cancerization in Lung Adenocarcinoma
Source: PLoS One. 2015 Feb 23;10(2):e0118132. doi: 10.1371/journal.pone.0118132 (PMC4338284; doi:10.1371/journal.pone.0118132)
Supplement: S6 Fig — (DOCX) [file pone.0118132.s006.docx]

**S6
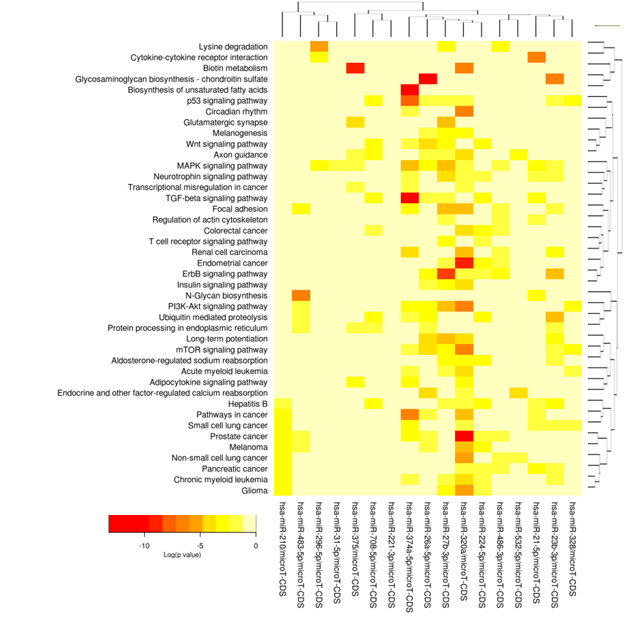
 Figure. Hierarchical heatmap of significant KEGG pathway based on differentiatially expressed miRNA in the peripheral airway field of cancerization with *micro-T-CDS prediction***. Using DIANA-mirPath with ***micro-T-CDS prediction***, hierarchial heatmap was generated with complete linkage clustering method and squared Euclidean distance measure. TOP 5 KEGG pathways were MAPk signaling pathway, PI3k-Akt signaling pathway, Focal adhesion, Pathways in cancer, and TGF-beta signaling pathways.
