# Supplementary material for: Molecular Characterization of the Peripheral Airway Field of Cancerization in Lung Adenocarcinoma
Source: PLoS One. 2015 Feb 23;10(2):e0118132. doi: 10.1371/journal.pone.0118132 (PMC4338284; doi:10.1371/journal.pone.0118132)

**S7 Figure. Correlation plot of RT-PCR vs. TaqMan array platform.** Plots of individual subjects show good correlation between RT-PCR data and TaqMan array data. Statistical calculation with Pearson’s correlation.


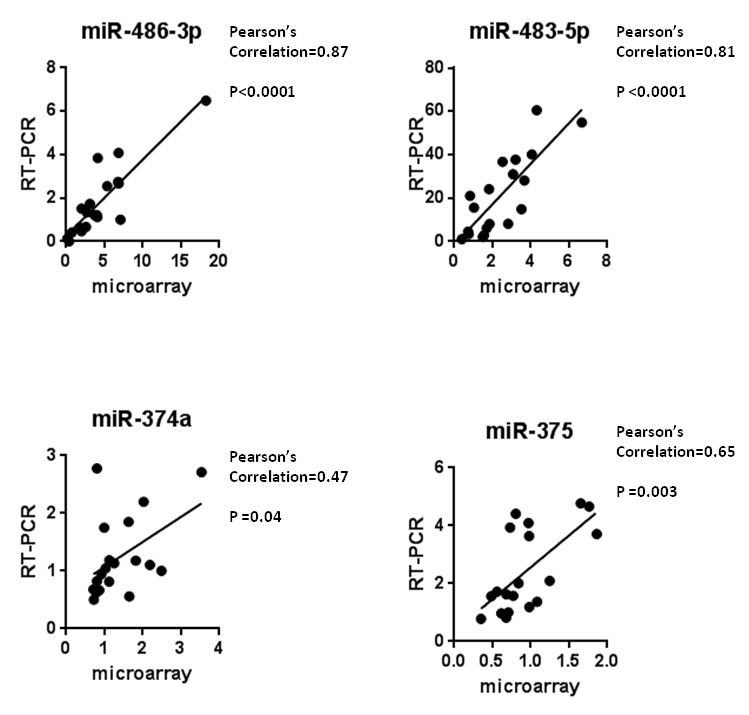

Supplement: S7 Fig — (DOCX) [file pone.0118132.s007.docx]
