# Supplementary material for: Molecular Characterization of the Peripheral Airway Field of Cancerization in Lung Adenocarcinoma
Source: PLoS One. 2015 Feb 23;10(2):e0118132. doi: 10.1371/journal.pone.0118132 (PMC4338284; doi:10.1371/journal.pone.0118132)
Supplement: S8 Fig — (DOCX) [file pone.0118132.s008.docx]

**S8 Figure. Molecular Signatures in Peripheral airway Field of Cancerization.** Support vector machines with leave-one-out cross validation (LOOCV) protocol was used to generate mRNA and miRNA molecular signatures in the field of cancerization which could be used to differentiate cancer patients from control smokers. **A)** Using mRNA (FDR<0.15 and |FC|>1.5 applied by LOOCV), a receiver operating characteristic (ROC) curve with area under the curve (AUC) of 0.92 was generated. **B)** Using miRNA (FDR<0.15 and |FC|>1.5 applied by LOOCV), a ROC curve with AUC of 0.94 was generated. **C)** The top 15 mRNAs most frequently selected during cross validation by LOOCV. **D)** The top 15 miRNAs most frequently selected during cross validation by LOOCV.


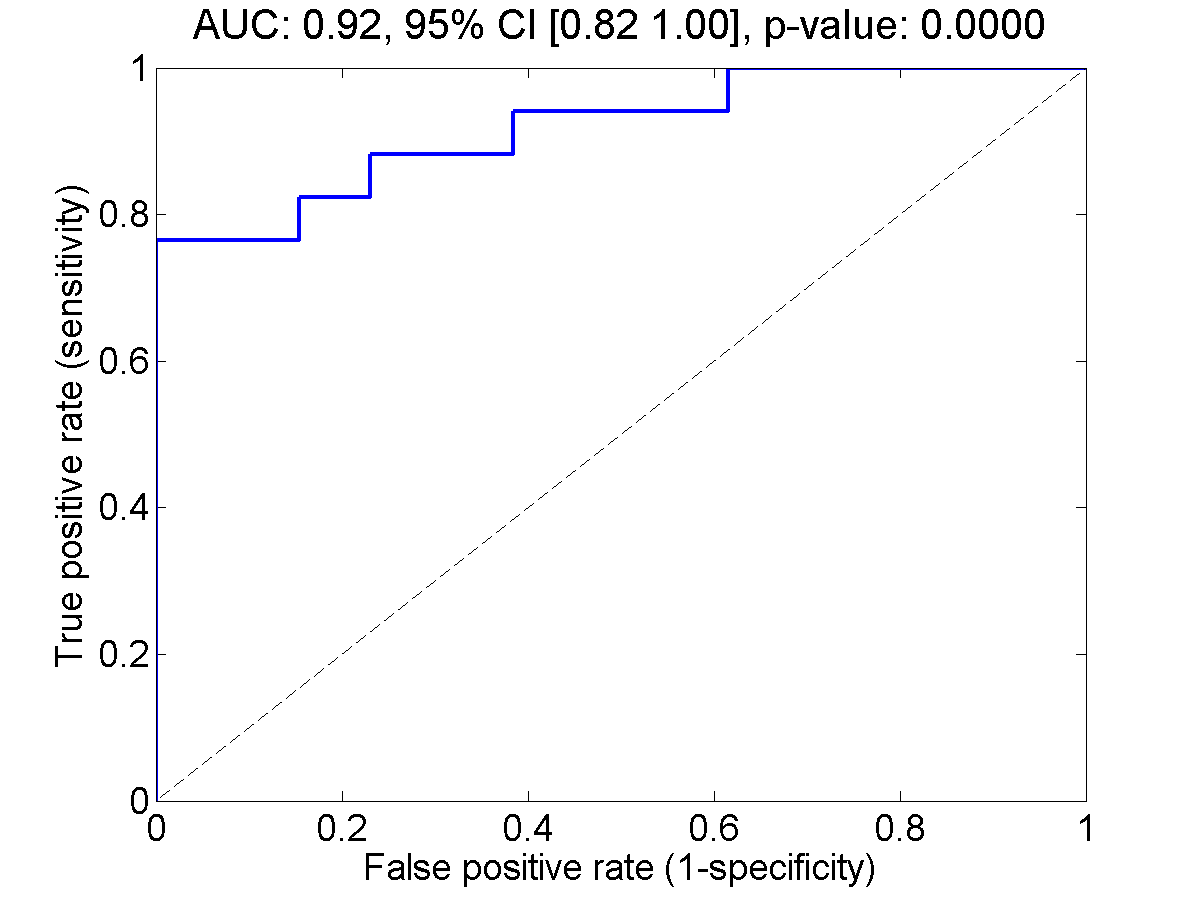

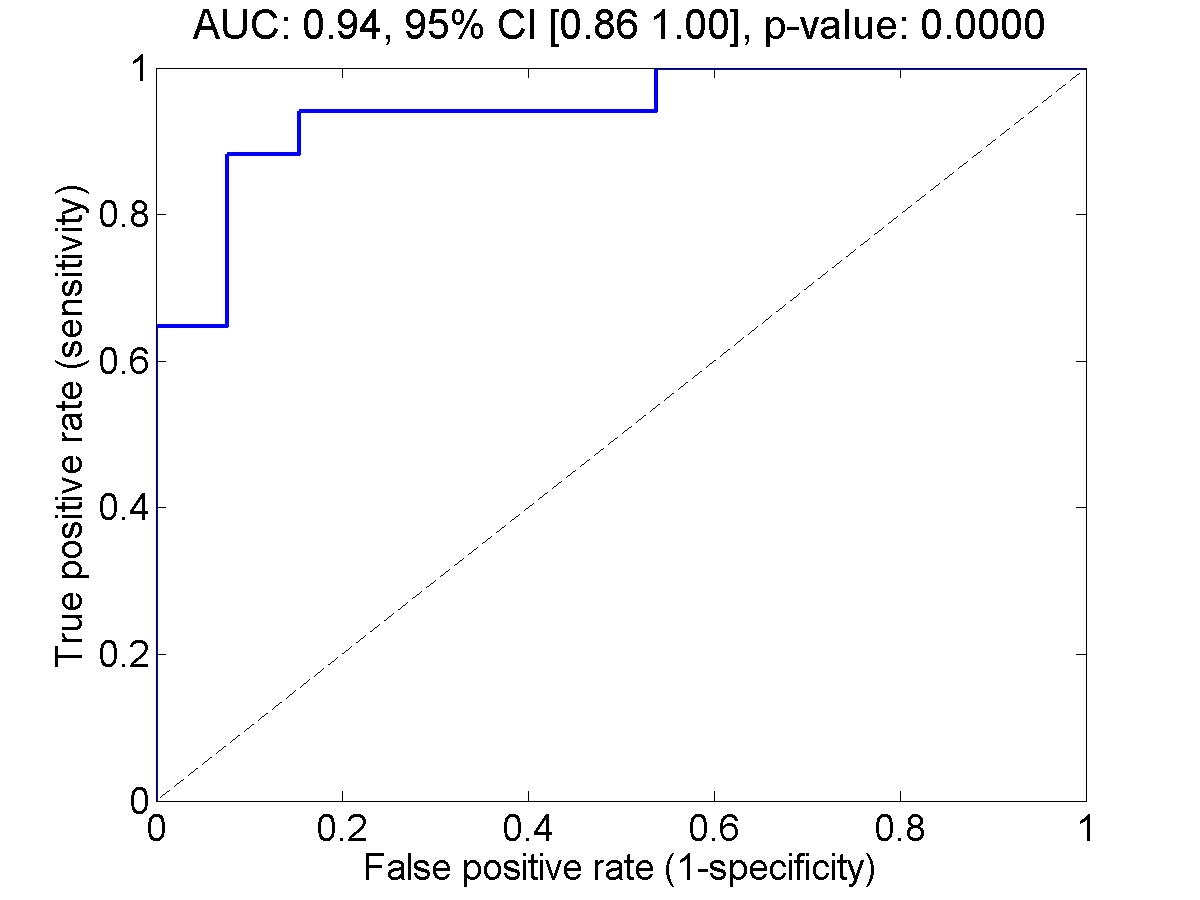


mRNA

miRNA

AUC=0.92

P<0.0001

AUC=0.94

P<0.0001

A

B

**C D**

| **Probe** | **Gene symbol** | **Frequency of selection by LOOCV** |  | **miRNA** | **Frequency of selection by LOOCV** |
| --- | --- | --- | --- | --- | --- |
| 201626_at | **INSIG1** | 100% |  | **hsa-let-7b** | 100.0% |
| 201627_s_at | **INSIG1** | 100% |  | **hsa-miR-140-3p** | 100.0% |
| 212724_at | **RND3** | 100% |  | **hsa-miR-221** | 100.0% |
| 235085_at | **SGK223** | 100% |  | **hsa-miR-23b** | 100.0% |
| 204260_at | **CHGB** | 96.7% |  | **hsa-miR-296-5p** | 100.0% |
| 205807_s_at | **TUFT1** | 73.3% |  | **hsa-miR-328** | 100.0% |
| 238987_at | **B4GALT1** | 73.3% |  | **hsa-miR-374a** | 100.0% |
| 230127_at | --- | 63.3% |  | **hsa-miR-375** | 100.0% |
| 204697_s_at | **CHGA** | 53.3% |  | **hsa-miR-483-5p** | 100.0% |
| 235739_at | **NR4A2** | 53.3% |  | **hsa-miR-486-3p** | 100.0% |
| 203002_at | **AMOTL2** | 50.0% |  | **hsa-miR-199a-3p** | 96.7% |
| 217561_at | **CALCA** | 50.0% |  | **hsa-miR-21** | 96.7% |
| 204455_at | **DST** | 40.0% |  | **hsa-miR-224** | 96.7% |
| 209988_s_at | **ASCL1** | 40.0% |  | **hsa-miR-320** | 96.7% |
| 242189_at | **HOMER1** | 40.0% |  | **hsa-miR-374b** | 96.7% |
