# Supplementary material for: Molecular Characterization of the Peripheral Airway Field of Cancerization in Lung Adenocarcinoma
Source: PLoS One. 2015 Feb 23;10(2):e0118132. doi: 10.1371/journal.pone.0118132 (PMC4338284; doi:10.1371/journal.pone.0118132)
Supplement: S1 Table — (DOCX) [file pone.0118132.s009.docx]

**S1 Table. Differentially expressed genes with Affymetrix Gene Chip HG U133 Plus 2.0, FDR <0.1**

| **probeset** | **FDR** | **t-test pvalue** | **Regulation** | **FC abs** | **FC** | **Log2FC** | **GeneSymbol** |
| --- | --- | --- | --- | --- | --- | --- | --- |
| 204260_at_ | 0.05 | 8.8E-06 | **down** | 1.9 | -1.9 | -0.9 | CHGB |
| 238987_at_ | 0.05 | 8.8E-06 | **down** | 2.1 | -2.1 | -1.0 | B4GALT1 |
| 218937_at_ | 0.07 | 1.4E-05 | **down** | 1.3 | -1.3 | -0.3 | ZSCAN32 |
| 201576_s_at | 0.07 | 1.7E-05 | **down** | 1.3 | -1.3 | -0.4 | GLB1 |
| 209988_s_at | 0.07 | 2.3E-05 | **down** | 2.8 | -2.8 | -1.5 | ASCL1 |
| 203273_s_at | 0.07 | 2.2E-05 | **down** | 1.3 | -1.3 | -0.4 | TUSC2 |
| 218480_at_ | 0.07 | 2.3E-05 | **down** | 1.4 | -1.4 | -0.5 | AGBL5 |
| 235739_at_ | 0.08 | 2.5E-05 | **down** | 2.5 | -2.5 | -1.3 |  |
| 217561_at_ | 0.09 | 3.3E-05 | **down** | 2.6 | -2.6 | -1.4 | CALCA |
| 225718_at_ | 0.09 | 3.3E-05 | **down** | 1.4 | -1.4 | -0.5 | KIAA1715 |
| 203277_at_ | 0.10 | 3.7E-05 | **down** | 1.4 | -1.4 | -0.5 | DFFA |
| 201797_s_at | 0.10 | 6.3E-05 | **down** | 1.4 | -1.4 | -0.5 | VARS |
| 204697_s_at | 0.10 | 5.1E-05 | **down** | 1.8 | -1.8 | -0.8 | CHGA |
| 242666_at_ | 0.10 | 6.0E-05 | **down** | 1.4 | -1.4 | -0.5 | OTTHUMG00000151249///RP3-400N23.6 |
| 202961_s_at | 0.10 | 6.2E-05 | **down** | 1.2 | -1.2 | -0.3 | ATP5J2 |
| 203924_at_ | 0.10 | 5.8E-05 | **down** | 1.2 | -1.2 | -0.3 | GSTA1 |
| 205059_s_at | 0.10 | 6.5E-05 | **down** | 1.4 | -1.4 | -0.4 | IDUA |
| 212782_x_at | 0.10 | 6.2E-05 | **down** | 1.2 | -1.2 | -0.3 | POLR2J |
| 222671_s_at | 0.10 | 5.3E-05 | **down** | 1.3 | -1.3 | -0.4 | JMJD4 |
| 1554558_at_ | 0.10 | 6.0E-05 | **down** | 1.4 | -1.4 | -0.5 | DCAF5 |
| 224972_at_ | 0.10 | 7.0E-05 | **down** | 1.2 | -1.2 | -0.3 | ROMO1 |
| 1555781_at_ | 0.10 | 7.7E-05 | **down** | 1.4 | -1.4 | -0.5 | PQLC2 |
| 206457_s_at | 0.10 | 1.1E-04 | **down** | 1.9 | -1.9 | -0.9 | DIO1 |
| 223479_s_at | 0.10 | 1.1E-04 | **down** | 1.7 | -1.7 | -0.7 | CHCHD5 |
| 228667_at_ | 0.10 | 9.9E-05 | **down** | 1.6 | -1.6 | -0.7 | AGPAT4 |
| 1552330_at_ | 0.10 | 9.1E-05 | **down** | 1.5 | -1.5 | -0.6 | CENPBD1 |
| 230931_at_ | 0.10 | 9.4E-05 | **down** | 1.4 | -1.4 | -0.5 | PLG |
| 201735_s_at | 0.10 | 9.5E-05 | **down** | 1.3 | -1.3 | -0.4 | CLCN3 |
| 204925_at_ | 0.10 | 9.0E-05 | **down** | 1.4 | -1.4 | -0.5 | CTNS |
| 209364_at_ | 0.10 | 1.1E-04 | **down** | 1.3 | -1.3 | -0.4 | BAD |
| 212798_s_at | 0.10 | 8.5E-05 | **down** | 1.3 | -1.3 | -0.4 | ANKMY2 |
| 213190_at_ | 0.10 | 8.4E-05 | **down** | 1.4 | -1.4 | -0.5 | COG7 |
| 218388_at_ | 0.10 | 9.1E-05 | **down** | 1.2 | -1.2 | -0.3 | PGLS |
| 227159_at_ | 0.10 | 1.1E-04 | **down** | 1.3 | -1.3 | -0.4 | GHDC |
| 227442_at_ | 0.10 | 1.0E-04 | **down** | 1.3 | -1.3 | -0.3 | COX18 |
| 41047_at_ | 0.10 | 1.2E-04 | **down** | 1.3 | -1.3 | -0.4 | C9orf16 |
| 204121_at_ | 0.10 | 1.2E-04 | **down** | 1.4 | -1.4 | -0.5 | GADD45G |
| 219953_s_at | 0.10 | 1.2E-04 | **down** | 1.3 | -1.3 | -0.3 | AKIP1 |
| 201626_at_ | 0.01 | 9.5E-08 | **up** | 1.7 | 1.7 | 0.8 | INSIG1 |
| 235085_at_ | 0.01 | 4.7E-07 | **up** | 1.6 | 1.6 | 0.6 | SGK223 |
| 212724_at_ | 0.02 | 8.9E-07 | **up** | 1.6 | 1.6 | 0.7 | RND3 |
| 201627_s_at | 0.03 | 1.9E-06 | **up** | 1.7 | 1.7 | 0.8 | INSIG1 |
| 204646_at_ | 0.04 | 3.9E-06 | **up** | 1.4 | 1.4 | 0.5 | DPYD |
| 205807_s_at | 0.05 | 5.3E-06 | **up** | 2.4 | 2.4 | 1.3 | TUFT1 |
| 230127_at_ | 0.05 | 6.9E-06 | **up** | 2.6 | 2.6 | 1.4 | OTTHUMG00000184015///RP6-99M1.2 |
| 1561857_at_ | 0.05 | 1.0E-05 | **up** | 1.2 | 1.2 | 0.2 |  |
| 203002_at_ | 0.07 | 1.8E-05 | **up** | 1.8 | 1.8 | 0.8 | AMOTL2 |
| 225895_at_ | 0.07 | 2.1E-05 | **up** | 2.1 | 2.1 | 1.1 | SYNPO2 |
| 242189_at_ | 0.10 | 4.2E-05 | **up** | 1.5 | 1.5 | 0.6 |  |
| 202068_s_at | 0.10 | 6.6E-05 | **up** | 1.5 | 1.5 | 0.6 | LDLR |
| 204455_at_ | 0.10 | 5.6E-05 | **up** | 3.2 | 3.2 | 1.7 | DST |
| 236307_at_ | 0.10 | 4.3E-05 | **up** | 1.8 | 1.8 | 0.8 |  |
| 215765_at_ | 0.10 | 6.1E-05 | **up** | 1.3 | 1.3 | 0.4 | LRRC41 |
| 1562777_at_ | 0.10 | 5.7E-05 | **up** | 1.2 | 1.2 | 0.3 | ERV3-1 |
| 209210_s_at | 0.10 | 7.0E-05 | **up** | 2.0 | 2.0 | 1.0 | FERMT2 |
| 230398_at_ | 0.10 | 7.3E-05 | **up** | 1.6 | 1.6 | 0.7 | TNS4 |
| 216918_s_at | 0.10 | 7.8E-05 | **up** | 2.4 | 2.4 | 1.3 | DST |
| 209824_s_at | 0.10 | 7.6E-05 | **up** | 1.6 | 1.6 | 0.7 | ARNTL |
| 225720_at_ | 0.10 | 1.1E-04 | **up** | 1.5 | 1.5 | 0.6 | SYNPO2 |
| 232471_at_ | 0.10 | 1.0E-04 | **up** | 1.9 | 1.9 | 1.0 |  |
| 243974_at_ | 0.10 | 1.1E-04 | **up** | 1.4 | 1.4 | 0.5 | OTTHUMG00000184053///RP3-331H24.6 |
| 202693_s_at | 0.10 | 9.3E-05 | **up** | 1.4 | 1.4 | 0.5 | STK17A |
| 223577_x_at | 0.10 | 1.2E-04 | **up** | 1.4 | 1.4 | 0.5 | LOC100996467 |
| 202820_at_ | 0.10 | 1.2E-04 | **up** | 1.3 | 1.3 | 0.4 | AHR |
