# Supplementary material for: Molecular Characterization of the Peripheral Airway Field of Cancerization in Lung Adenocarcinoma
Source: PLoS One. 2015 Feb 23;10(2):e0118132. doi: 10.1371/journal.pone.0118132 (PMC4338284; doi:10.1371/journal.pone.0118132)
Supplement: S2 Table — (DOCX) [file pone.0118132.s010.docx]

**S2 Table. DAVID functional enrichment analysis**

| Category | Term | Fold Enrichment | P-value | Genes |
| --- | --- | --- | --- | --- |
| GOTERM_BP_FAT | GO:0006915~apoptosis | 3.9 | 0.01 | TNS4, DFFA, GADD45G, STK17A, BAD, AHR, PLG |
| GOTERM_BP_FAT | GO:0012501~programmed cell death | 3.9 | 0.01 | TNS4, DFFA, GADD45G, STK17A, BAD, AHR, PLG |
| GOTERM_BP_FAT | GO:0005984~disaccharide metabolic process | 133.1 | 0.01 | B4GALT1, IDUA |
| GOTERM_BP_FAT | GO:0008219~cell death | 3.3 | 0.02 | TNS4, DFFA, GADD45G, STK17A, BAD, AHR, PLG |
| GOTERM_BP_FAT | GO:0016265~death | 3.3 | 0.02 | TNS4, DFFA, GADD45G, STK17A, BAD, AHR, PLG |
| GOTERM_BP_FAT | GO:0043065~positive regulation of apoptosis | 3.9 | 0.03 | B4GALT1, DFFA, STK17A, BAD, PLG |
| GOTERM_BP_FAT | GO:0043068~positive regulation of programmed cell death | 3.9 | 0.04 | B4GALT1, DFFA, STK17A, BAD, PLG |
| GOTERM_BP_FAT | GO:0010942~positive regulation of cell death | 3.9 | 0.04 | B4GALT1, DFFA, STK17A, BAD, PLG |
| GOTERM_BP_FAT | GO:0008283~cell proliferation | 3.9 | 0.04 | CALCA, TUSC2, ASCL1, INSIG1, BAD |
|  |  |  |  |  |
| GOTERM_CC_FAT | GO:0016323~basolateral plasma membrane | 6.6 | 0.02 | TNS4, B4GALT1, FERMT2, DST |
| GOTERM_CC_FAT | GO:0030055~cell-substrate junction | 9.0 | 0.04 | TNS4, FERMT2, DST |
|  |  |  |  |  |
| GOTERM_MF_FAT | GO:0051879~Hsp90 protein binding | 69.1 | 0.03 | ARNTL, AHR |
|  |  |  |  |  |
| SP_PIR_KEYWORDS | Apoptosis | 5.0 | 0.02 | TNS4, DFFA, GADD45G, STK17A, BAD |
| SP_PIR_KEYWORDS | cleavage on pair of basic residues | 5.7 | 0.03 | CALCA, CHGA, CHGB, PLG |
| SP_PIR_KEYWORDS | amidated carboxyl end | 43.6 | 0.04 | CALCA, CHGA |
| SP_PIR_KEYWORDS | congenital disorder of glycosylation | 35.3 | 0.05 | B4GALT1, COG7 |
