# Supplementary material for: Molecular Characterization of the Peripheral Airway Field of Cancerization in Lung Adenocarcinoma
Source: PLoS One. 2015 Feb 23;10(2):e0118132. doi: 10.1371/journal.pone.0118132 (PMC4338284; doi:10.1371/journal.pone.0118132)
Supplement: S3 Table — (DOCX) [file pone.0118132.s011.docx]

**S3 Table. Gene Set Enrichment Analysis (GSEA) with MSigDB C4 cancer gene neighborhoods (CGN) collection. FDR <0.1**

| **Gene Set C4 CGN** | **Size** | **ES** | **NES** | **NOM p-val** | **FDR q-val** |
| --- | --- | --- | --- | --- | --- |
| [MORF_DAP](http://www.broadinstitute.org/gsea/msigdb/cards/MORF_DAP) | 71 | 0.62 | 1.97 | 0.000 | 0.06 |
| [MORF_AP2M1](http://www.broadinstitute.org/gsea/msigdb/cards/MORF_AP2M1) | 193 | 0.54 | 1.93 | 0.004 | 0.06 |
| [MORF_MAP2K2](http://www.broadinstitute.org/gsea/msigdb/cards/MORF_MAP2K2) | 121 | 0.59 | 1.89 | 0.002 | 0.08 |
| [MORF_DDB1](http://www.broadinstitute.org/gsea/msigdb/cards/MORF_DDB1) | 214 | 0.49 | 1.80 | 0.004 | 0.09 |
| [MORF_CSNK2B](http://www.broadinstitute.org/gsea/msigdb/cards/MORF_CSNK2B) | 251 | 0.52 | 1.84 | 0.010 | 0.09 |
| [MORF_NME2](http://www.broadinstitute.org/gsea/msigdb/cards/MORF_NME2) | 140 | 0.48 | 1.82 | 0.025 | 0.09 |
| [MORF_PSMC1](http://www.broadinstitute.org/gsea/msigdb/cards/MORF_PSMC1) | 160 | 0.52 | 1.80 | 0.010 | 0.09 |
| [MORF_RAN](http://www.broadinstitute.org/gsea/msigdb/cards/MORF_RAN) | 237 | 0.47 | 1.81 | 0.026 | 0.10 |
| [MORF_PPP1CA](http://www.broadinstitute.org/gsea/msigdb/cards/MORF_PPP1CA) | 146 | 0.52 | 1.83 | 0.016 | 0.10 |
| [MORF_PHB](http://www.broadinstitute.org/gsea/msigdb/cards/MORF_PHB) | 110 | 0.53 | 1.85 | 0.006 | 0.10 |
