# Supplementary material for: Molecular Characterization of the Peripheral Airway Field of Cancerization in Lung Adenocarcinoma
Source: PLoS One. 2015 Feb 23;10(2):e0118132. doi: 10.1371/journal.pone.0118132 (PMC4338284; doi:10.1371/journal.pone.0118132)
Supplement: S4 Table — (DOCX) [file pone.0118132.s012.docx]

**S4 Table. Gene Set Enrichment Analysis (GSEA) with MSigDB C4 cancer gene modules (CM) collection. FDR <0.1**

| **Gene Set C4 CM** | **SIZE** | **ES** | **NES** | **NOM p-val** | **FDR q-val** |
| --- | --- | --- | --- | --- | --- |
| [MODULE_22](http://www.broadinstitute.org/gsea/msigdb/cards/MODULE_22) | 44 | 0.62 | 1.80 | 0.004 | 0.04 |
| [MODULE_159](http://www.broadinstitute.org/gsea/msigdb/cards/MODULE_159) | 66 | 0.48 | 1.80 | 0.002 | 0.04 |
| [MODULE_151](http://www.broadinstitute.org/gsea/msigdb/cards/MODULE_151) | 274 | 0.44 | 1.81 | 0.012 | 0.04 |
| [MODULE_116](http://www.broadinstitute.org/gsea/msigdb/cards/MODULE_116) | 28 | 0.61 | 1.85 | 0.002 | 0.04 |
| [MODULE_83](http://www.broadinstitute.org/gsea/msigdb/cards/MODULE_83) | 264 | 0.46 | 1.81 | 0.013 | 0.04 |
| [MODULE_299](http://www.broadinstitute.org/gsea/msigdb/cards/MODULE_299) | 33 | 0.64 | 1.91 | 0.000 | 0.05 |
| [MODULE_306](http://www.broadinstitute.org/gsea/msigdb/cards/MODULE_306) | 25 | 0.62 | 1.77 | 0.016 | 0.05 |
| [MODULE_114](http://www.broadinstitute.org/gsea/msigdb/cards/MODULE_114) | 289 | 0.45 | 1.85 | 0.010 | 0.05 |
| [MODULE_62](http://www.broadinstitute.org/gsea/msigdb/cards/MODULE_62) | 87 | 0.58 | 1.81 | 0.008 | 0.05 |
| [MODULE_93](http://www.broadinstitute.org/gsea/msigdb/cards/MODULE_93) | 175 | 0.48 | 1.81 | 0.000 | 0.05 |
| [MODULE_355](http://www.broadinstitute.org/gsea/msigdb/cards/MODULE_355) | 28 | 0.59 | 1.85 | 0.004 | 0.06 |
| [MODULE_43](http://www.broadinstitute.org/gsea/msigdb/cards/MODULE_43) | 96 | 0.48 | 1.86 | 0.000 | 0.07 |
| [MODULE_152](http://www.broadinstitute.org/gsea/msigdb/cards/MODULE_152) | 118 | 0.58 | 1.92 | 0.002 | 0.08 |
| [MODULE_77](http://www.broadinstitute.org/gsea/msigdb/cards/MODULE_77) | 27 | 0.67 | 1.69 | 0.006 | 0.10 |
