# Supplementary material for: Molecular Characterization of the Peripheral Airway Field of Cancerization in Lung Adenocarcinoma
Source: PLoS One. 2015 Feb 23;10(2):e0118132. doi: 10.1371/journal.pone.0118132 (PMC4338284; doi:10.1371/journal.pone.0118132)
Supplement: S5 Table — (DOCX) [file pone.0118132.s013.docx]

**S5 Table. Top differentially expressed microRNAs with TaqMan miRNA Array**

| Down-regulated | | | |
| --- | --- | --- | --- |
| **miRNA** | **t-test p-value** | **Benjamini FDR** | **Fold Change** |
| hsa-miR-224 | 0.001 | 0.04 | 0.43 |
| hsa-miR-708 | 0.001 | 0.04 | 0.43 |
| hsa-miR-221 | 0.001 | 0.04 | 0.40 |
| hsa-miR-328 | 0.001 | 0.05 | 0.46 |
| hsa-miR-375 | 0.002 | 0.05 | 0.31 |
| hsa-miR-23b | 0.002 | 0.05 | 0.34 |
| hsa-miR-21 | 0.003 | 0.07 | 0.41 |
| hsa-miR-27b | 0.004 | 0.07 | 0.45 |
| hsa-miR-532-5p | 0.004 | 0.07 | 0.63 |
| hsa-miR-296-5p | 0.005 | 0.09 | 0.27 |
| hsa-miR-26a | 0.005 | 0.09 | 0.60 |
| hsa-miR-31 | 0.007 | 0.10 | 0.67 |

| Up-regulated | | | |
| --- | --- | --- | --- |
| **miRNA** | **t-test p-value** | **Benjamini FDR** | **Fold Change** |
| hsa-miR-483-5p | <0.0001 | 0.04 | 4.1 |
| hsa-miR-374a | 0.001 | 0.05 | 1.9 |
| hsa-miR-486-3p | 0.001 | 0.05 | 4.8 |
| hsa-miR-210 | 0.003 | 0.07 | 1.5 |
| hsa-miR-320 | 0.004 | 0.07 | 1.6 |
