# Supplementary material for: Molecular Characterization of the Peripheral Airway Field of Cancerization in Lung Adenocarcinoma
Source: PLoS One. 2015 Feb 23;10(2):e0118132. doi: 10.1371/journal.pone.0118132 (PMC4338284; doi:10.1371/journal.pone.0118132)
Supplement: S6 Table — (DOCX) [file pone.0118132.s014.docx]

**S6 Table. Integrative negatively correlated mRNA-miRNA pairings base on TargetScan.**

|  | miRNA | Fold Change | mRNA | Fold Change | Pearson's correlation |
| --- | --- | --- | --- | --- | --- |
| **Down-regulated miRNA** | hsa-miR-221 | -1.3 | INSIG1 | 1.7 | -0.67 |
|  | hsa-miR-27b | -1.1 | SYNPO2 | 2.1 | -0.61 |
|  | hsa-miR-328 | -1.1 | PPP1R12B | 1.4 | -0.57 |
|  | hsa-miR-27b | -1.1 | LDLR | 1.5 | -0.47 |
|  | hsa-miR-328 | -1.1 | FERMT2 | 2.0 | -0.42 |
|  | hsa-miR-328 | -1.1 | SYNPO2 | 2.1 | -0.42 |
|  | hsa-miR-328 | -1.1 | TNS4 | 1.6 | -0.42 |
|  | hsa-miR-224 | -1.2 | SYNPO2 | 2.1 | -0.41 |
|  | hsa-miR-708 | -1.2 | AMOTL2 | 1.8 | -0.41 |
|  | hsa-miR-221 | -1.3 | FERMT2 | 2.0 | -0.40 |
|  | hsa-miR-221 | -1.3 | IFRD1 | 1.6 | -0.40 |
|  | hsa-miR-34c-5p | -0.8 | DPYD | 1.4 | -0.40 |
|  | hsa-miR-34c-5p | -0.8 | TUFT1 | 2.4 | -0.40 |
|  | hsa-miR-30c | -0.5 | IFRD1 | 1.6 | -0.40 |
|  | hsa-miR-30c | -0.5 | LDLR | 1.5 | -0.40 |
|  | hsa-miR-296-5p | -1.9 | EMP1 | 2.2 | -0.36 |
|  | hsa-miR-23b | -1.6 | SYNPO2 | 2.1 | -0.35 |
|  | hsa-miR-23b | -1.6 | ZNF267 | 1.4 | -0.35 |
|  | hsa-miR-23b | -1.6 | PDE7A | 1.4 | -0.35 |
|  | hsa-miR-27b | -1.1 | AMOTL2 | 1.8 | -0.34 |
|  | hsa-miR-21 | -1.3 | FAM3C | 1.7 | -0.28 |
|  | hsa-miR-708 | -1.2 | DUSP4 | 1.5 | -0.27 |
|  | hsa-miR-708 | -1.2 | TNS4 | 1.6 | -0.27 |
|  | hsa-miR-26a | -0.7 | DUSP4 | 1.5 | -0.27 |
|  | hsa-miR-26a | -0.7 | SYNPO2 | 2.1 | -0.27 |
|  | hsa-miR-31 | -0.6 | DPYD | 1.4 | -0.27 |
|  | hsa-miR-31 | -0.6 | CD55 | 1.8 | -0.27 |
|  | hsa-miR-31 | -0.6 | EMP1 | 2.2 | -0.27 |
|  | hsa-miR-31 | -0.6 | HERPUD2 | 1.2 | -0.27 |
|  | hsa-miR-30c | -0.5 | PRKAR1A | 1.3 | -0.27 |
|  | hsa-miR-30c | -0.5 | STK17B | 1.9 | -0.27 |
|  | hsa-miR-296-5p | -1.9 | PPP1R12B | 1.4 | -0.22 |
|  | hsa-miR-23b | -1.6 | USP31 | 1.3 | -0.21 |
|  | hsa-miR-532-5p | -0.7 | STK17B | 1.9 | -0.21 |
|  | hsa-miR-27b | -1.1 | USP31 | 1.3 | -0.20 |
|  | hsa-miR-375 | -1.7 | AHR | 1.3 | -0.15 |
|  | hsa-miR-21 | -1.3 | PER2 | 1.4 | -0.14 |
|  | hsa-miR-224 | -1.2 | FHL2 | 1.9 | -0.14 |
|  | hsa-miR-205 | -0.7 | DPYD | 1.4 | -0.14 |
|  | hsa-miR-132 | -0.7 | PDE7A | 1.4 | -0.14 |
|  | hsa-miR-34c-5p | -0.8 | AMOTL2 | 1.8 | -0.13 |
|  | hsa-miR-34c-5p | -0.8 | PER2 | 1.4 | -0.13 |
|  | hsa-miR-34c-5p | -0.8 | USP31 | 1.3 | -0.13 |
|  | hsa-miR-31 | -0.6 | FHL2 | 1.9 | -0.13 |
|  | hsa-miR-30c | -0.5 | AMOTL2 | 1.8 | -0.13 |
|  | hsa-miR-30c | -0.5 | PDE7A | 1.4 | -0.13 |
|  | hsa-miR-30c | -0.5 | PER2 | 1.4 | -0.13 |
|  | hsa-miR-296-5p | -1.9 | LDLR | 1.5 | -0.07 |
|  | hsa-miR-532-5p | -0.7 | B2M | 1.2 | -0.07 |
|  | hsa-miR-34c-5p | -0.8 | DUSP4 | 1.5 | 0.00 |
|  | miRNA | Fold Change | mRNA | Fold Change | Pearson's correlation |
| **Up-regulated miRNA** | hsa-miR-486-3p | 2.3 | TBC1D24 | -1.3 | -0.71 |
|  | hsa-miR-483-5p | 2.0 | CLCN3 | -1.3 | -0.63 |
|  | hsa-miR-320 | 0.7 | JMJD4 | -1.3 | -0.60 |
|  | hsa-miR-320 | 0.7 | ST3GAL1 | -1.3 | -0.60 |
|  | hsa-miR-199a-3p | 1.0 | COG7 | -1.4 | -0.57 |
|  | hsa-miR-486-3p | 2.3 | ZFP90 | -1.5 | -0.57 |
|  | hsa-miR-486-3p | 2.3 | DAG1 | -1.2 | -0.57 |
|  | hsa-miR-486-3p | 2.3 | MAP6 | -1.4 | -0.57 |
|  | hsa-miR-486-3p | 2.3 | MEN1 | -1.4 | -0.57 |
|  | hsa-miR-483-5p | 2.0 | MCUR1 | -1.2 | -0.54 |
|  | hsa-miR-483-5p | 2.0 | EMC10 | -1.3 | -0.54 |
|  | hsa-let-7b | 1.2 | IDH2 | -1.4 | -0.54 |
|  | hsa-miR-140-3p | 1.0 | TMEM70 | -1.4 | -0.48 |
|  | hsa-miR-140-3p | 1.0 | CTNNA1 | -1.2 | -0.48 |
|  | hsa-miR-486-3p | 2.3 | MRS2 | -1.5 | -0.47 |
|  | hsa-let-7b | 1.2 | MRS2 | -1.5 | -0.46 |
|  | hsa-miR-486-3p | 2.3 | FBXL18 | -1.4 | -0.42 |
|  | hsa-miR-486-3p | 2.3 | DFFA | -1.4 | -0.42 |
|  | hsa-miR-486-3p | 2.3 | SMG5 | -1.2 | -0.42 |
|  | hsa-miR-486-3p | 2.3 | DCAF7 | -1.4 | -0.42 |
|  | hsa-miR-486-3p | 2.3 | CHCHD5 | -1.7 | -0.42 |
|  | hsa-miR-486-3p | 2.3 | GHDC | -1.3 | -0.42 |
|  | hsa-miR-483-5p | 2.0 | COPA | -1.4 | -0.41 |
|  | hsa-miR-483-5p | 2.0 | FAM83A | -1.4 | -0.41 |
|  | hsa-miR-483-5p | 2.0 | PLIN3 | -1.4 | -0.41 |
|  | hsa-let-7b | 1.2 | SREK1IP1 | -1.4 | -0.40 |
|  | hsa-let-7b | 1.2 | ST3GAL1 | -1.3 | -0.40 |
|  | hsa-miR-374a | 0.9 | ASCL1 | -2.8 | -0.35 |
|  | hsa-miR-140-3p | 1.0 | VCPIP1 | -1.3 | -0.35 |
|  | hsa-miR-140-3p | 1.0 | DIAPH2 | -1.5 | -0.35 |
|  | hsa-miR-140-3p | 1.0 | AGPAT4 | -1.6 | -0.35 |
|  | hsa-miR-140-3p | 1.0 | USP14 | -1.4 | -0.35 |
|  | hsa-let-7b | 1.2 | MLXIP | -1.2 | -0.34 |
|  | hsa-miR-320 | 0.7 | ATP2A3 | -1.4 | -0.33 |
|  | hsa-miR-320 | 0.7 | HEY1 | -1.4 | -0.33 |
|  | hsa-miR-320 | 0.7 | MSI2 | -1.2 | -0.33 |
|  | hsa-miR-320 | 0.7 | SREK1IP1 | -1.4 | -0.33 |
|  | hsa-miR-320 | 0.7 | USP14 | -1.4 | -0.33 |
|  | hsa-miR-320 | 0.7 | CHGA | -1.8 | -0.33 |
|  | hsa-miR-486-3p | 2.3 | TM9SF4 | -1.3 | -0.28 |
|  | hsa-miR-483-5p | 2.0 | CENPBD1 | -1.5 | -0.27 |
|  | hsa-miR-320 | 0.7 | KIAA1715 | -1.4 | -0.27 |
|  | hsa-miR-210 | 0.6 | ASCL1 | -2.8 | -0.27 |
|  | hsa-miR-210 | 0.6 | DCAF5 | -1.4 | -0.27 |
|  | hsa-let-7b | 1.2 | CTNS | -1.4 | -0.27 |
|  | hsa-let-7b | 1.2 | PARS2 | -1.3 | -0.27 |
|  | hsa-let-7b | 1.2 | VCPIP1 | -1.3 | -0.27 |
|  | hsa-miR-140-3p | 1.0 | HEY1 | -1.4 | -0.21 |
|  | hsa-miR-210 | 0.6 | SMG5 | -1.2 | -0.13 |
|  | hsa-let-7b | 1.2 | PQLC2 | -1.4 | -0.13 |
|  | hsa-miR-199a-3p | 1.0 | CD151 | -1.4 | 0.00 |
|  | hsa-let-7b | 1.2 | TUSC2 | -1.3 | 0.13 |
