# Supplementary material for: Molecular Characterization of the Peripheral Airway Field of Cancerization in Lung Adenocarcinoma
Source: PLoS One. 2015 Feb 23;10(2):e0118132. doi: 10.1371/journal.pone.0118132 (PMC4338284; doi:10.1371/journal.pone.0118132)
Supplement: S7 Table — (DOCX) [file pone.0118132.s015.docx]

**S7 Table. Transcriptomic profile comparison to Kadara et al. (GSE44077)**

|  |  | Peripheral Airway | | GSE44077 farthest airway (Kadara et al.)† | | | GSE44077 closest airway (Kadara et al.)† | | |
| --- | --- | --- | --- | --- | --- | --- | --- | --- | --- |
| Probe Set ID | Gene Symbol | Regulation | Log2 FC | Regulation | logFC | BH-FDR | Regulation | logFC | BH-FDR |
| 218480_at | AGBL5 | down | -0.5 | down | -0.9 | 7.9E-11 | up | 0.9 | 3.4E-16 |
| 228667_at | AGPAT4 | down | -0.7 | up | 0.7 | 5.8E-06 | down | -0.8 | 1.9E-07 |
| 202820_at | AHR | up | 0.4 | up | 0.3 | 4.3E-04 | down | -0.2 | 5.9E-03 |
| 219953_s_at | AKIP1 | down | -0.3 | up | 0.6 | 2.8E-06 | down | -0.3 | 2.2E-02 |
| 203002_at | AMOTL2 | up | 0.8 | up | 0.6 | 1.3E-05 | down | -0.6 | 1.4E-06 |
| 212798_s_at | ANKMY2 | down | -0.4 | down | -0.7 | 5.2E-08 | up | 0.6 | 4.9E-10 |
| 209824_s_at | ARNTL | up | 0.7 |  |  |  | down | -0.4 | 4.8E-03 |
| 209988_s_at | ASCL1 | down | -1.5 | down | -0.4 | 1.2E-03 | up | 0.5 | 1.1E-04 |
| 202961_s_at | ATP5J2 | down | -0.3 |  |  |  | up | 0.2 | 2.7E-02 |
| 238987_at | B4GALT1 | down | -1.0 |  |  |  |  |  |  |
| 209364_at | BAD | down | -0.4 | down | -0.1 | 2.9E-02 | up | 0.2 | 6.1E-06 |
| 41047_at | C9orf16 | down | -0.4 | up | 0.2 | 2.5E-03 |  |  |  |
| 217561_at | CALCA | down | -1.4 | down | -0.3 | 8.2E-04 | up | 0.4 | 6.0E-05 |
| 1552330_at | CENPBD1 | down | -0.6 |  |  |  |  |  |  |
| 223479_s_at | CHCHD5 | down | -0.7 |  |  |  | up | 0.1 | 1.2E-02 |
| 204697_s_at | CHGA | down | -0.8 | down | -0.2 | 1.0E-03 | up | 0.3 | 8.8E-03 |
| 204260_at | CHGB | down | -0.9 | down | -0.3 | 2.6E-04 | up | 0.5 | 1.0E-05 |
| 201735_s_at | CLCN3 | down | -0.4 | down | -0.8 | 9.2E-10 | up | 0.8 | 9.7E-11 |
| 213190_at | COG7 | down | -0.5 | down | -1.1 | 1.4E-13 | up | 1.0 | 2.7E-14 |
| 227442_at | COX18 | down | -0.3 |  |  |  |  |  |  |
| 204925_at | CTNS | down | -0.5 |  |  |  |  |  |  |
| 1554558_at | DCAF5 | down | -0.5 |  |  |  | down | -0.2 | 8.9E-04 |
| 203277_at | DFFA | down | -0.5 |  |  |  |  |  |  |
| 206457_s_at | DIO1 | down | -0.9 | down | -1.7 | 7.6E-13 | up | 1.6 | 4.4E-13 |
| 204646_at | DPYD | up | 0.5 | up | 0.8 | 1.4E-11 | down | -0.7 | 1.7E-12 |
| 204455_at | DST* | up | 1.7 | up | 0.8 | 3.7E-02 | down | -0.8 | 2.3E-06 |
| 1562777_at | ERV3-1 | up | 0.3 | up | 0.2 | 1.4E-05 | down | -0.3 | 3.7E-02 |
| 209209_s_at | FERMT2 | up | 0.7 | up | 0.9 | 6.7E-05 | down | -1.0 | 1.9E-05 |
| 204121_at | GADD45G | down | -0.5 | down | -0.4 | 2.7E-03 | up | 0.2 | 3.3E-02 |
| 227159_at | GHDC | down | -0.4 | up | 0.2 | 1.4E-02 | down | -0.2 | 5.6E-03 |
| 201576_s_at | GLB1 | down | -0.4 |  |  |  | down | -0.7 | 6.7E-08 |
| 203924_at | GSTA1 | down | -0.3 | down | -3.5 | 2.2E-12 | up | 3.2 | 3.0E-14 |
| 205059_s_at | IDUA | down | -0.4 | up | 0.1 | 4.4E-02 | down | -0.2 | 8.3E-03 |
| 201626_at | INSIG1 | up | 0.8 |  |  |  |  |  |  |
| 201627_s_at | INSIG1 | up | 0.8 |  |  |  |  |  |  |
| 222671_s_at | JMJD4 | down | -0.4 | down | -0.2 | 3.3E-04 | up | 0.2 | 3.5E-05 |
| 225718_at | KIAA1715 | down | -0.5 | up | 0.3 | 8.3E-05 | down | -0.2 | 3.4E-02 |
| 202068_s_at | LDLR | up | 0.6 |  |  |  | down | -0.4 | 3.1E-03 |
| 223577_x_at | LOC100996467 | up | 0.5 |  |  |  |  |  |  |
| 215765_at | LRRC41 | up | 0.4 | down | -0.1 | 3.6E-02 | up | 0.2 | 1.9E-04 |
| 218388_at | PGLS | down | -0.3 |  |  |  | up | 0.1 | 5.2E-02 |
| 230931_at | PLG | down | -0.5 | down | -0.6 | 2.0E-02 | up | 0.5 | 4.1E-02 |
| 212782_x_at | POLR2J | down | -0.3 |  |  |  | up | 0.1 | 3.8E-02 |
| 1555781_at | PQLC2 | down | -0.5 | up | 0.3 | 7.8E-07 | down | -0.2 | 2.3E-05 |
| 212724_at | RND3 | up | 0.7 | down | -0.4 | 3.8E-02 | up | 0.4 | 9.2E-02 |
| 224972_at | ROMO1 | down | -0.3 | down | -0.3 | 8.5E-03 | up | 0.5 | 1.1E-06 |
| 243974_at | RP3-331 | up | 0.5 |  |  |  |  |  |  |
| 242666_at | RP3-400N | down | -0.5 |  |  |  |  |  |  |
| 230127_at | RP6-99M1.2 | up | 1.4 |  |  |  |  |  |  |
| 235085_at | SGK223 | up | 0.6 | up | 0.3 | 7.7E-06 | down | -0.3 | 5.7E-06 |
| 202693_s_at | STK17A | up | 0.5 | down | -0.3 | 1.6E-02 | up | 0.3 | 4.0E-02 |
| 225895_at | SYNPO2* | up | 1.1 | down | -0.6 | 6.7E-03 | up | 0.4 | 8.9E-02 |
| 230398_at | TNS4 | up | 0.7 | down | -3.2 | 4.3E-20 | up | 3.3 | 1.3E-22 |
| 205807_s_at | TUFT1 | up | 1.3 | down | -0.6 | 9.5E-04 | up | 0.9 | 2.1E-05 |
| 203273_s_at | TUSC2 | down | -0.4 | down | -0.3 | 2.1E-03 | up | 0.3 | 7.9E-07 |
| 201797_s_at | VARS | down | -0.5 | up | 0.2 | 5.7E-03 | up | 0.2 | 1.5E-02 |
| 218937_at | ZSCAN32 | down | -0.3 | up | 0.2 | 1.50E-03 | down | -0.1 | 1.3E-02 |
| 1561857_at |  | up | 0.2 |  |  |  |  |  |  |
| 232471_at |  | up | 1.0 |  |  |  |  |  |  |
| 235739_at |  | down | -1.3 |  |  |  |  |  |  |
| 236307_at |  | up | 0.8 |  |  |  |  |  |  |
| 242189_at |  | up | 0.6 |  |  |  |  |  |  |
| * DST and SYNPO2 were detected by twice | | | | |  |  |  |  |  |
| † Statistics Benjamini-Hochberg FDR <0.1 | | | | |  |  |  |  |  |
