# Supplementary material for: Molecular Characterization of the Peripheral Airway Field of Cancerization in Lung Adenocarcinoma
Source: PLoS One. 2015 Feb 23;10(2):e0118132. doi: 10.1371/journal.pone.0118132 (PMC4338284; doi:10.1371/journal.pone.0118132)
Supplement: S8 Table — (DOCX) [file pone.0118132.s016.docx]

**S8 Table. Gene expressions comparison between peripheral airway epithelial cells and large airway epithelial cells (Spira et al. GSE4115)**

|  | Peripheral Airway | | Spira et al. Large Airway GSE4115 | |
| --- | --- | --- | --- | --- |
| **GeneSymbol** | **Regulation** | **Log2 FC** | **Regulation** | **Log FC** |
| B4GALT1 | down | -1.0 | down | -0.07 |
| ARNTL | up | 0.7 | down | -0.11 |
| BAD | down | -0.4 | down | -0.16 |
| CALCA | down | -1.4 | down | -0.15 |
| CLCN3 | down | -0.4 | down | -0.16 |
| DFFA | down | -0.5 | down | -0.03 |
| DIO1 | down | -0.9 | up | 0.06 |
| DST | up | 1.7 | up | 0.09 |
| IDUA | down | -0.4 | down | -0.04 |
| INSIG1 | up | 0.8 | up | 0.05 |
| STK17A | up | 0.5 | up | 0.04 |
| TUSC2 | down | -0.4 | up | 0.04 |
| ZSCAN32 | down | -0.3 | up | 0.03 |
|  |  |  |  |  |

***Statistics Benjamini-Hochberg FDR <0.05**

**Highlighted- expression level in similar direction**
