# Supplementary material for: Molecular Characterization of the Peripheral Airway Field of Cancerization in Lung Adenocarcinoma
Source: PLoS One. 2015 Feb 23;10(2):e0118132. doi: 10.1371/journal.pone.0118132 (PMC4338284; doi:10.1371/journal.pone.0118132)
Supplement: S1 Methods — (DOCX) [file pone.0118132.s017.docx]

**Molecular Characterization of the Peripheral Airway Field of Cancerization in Lung Adenocarcinoma**

Jun-Chieh J Tsay¹, Zhiguo Li², Ting-An Yie¹, Feng Wu³, Leopoldo Segal¹, Alissa K Greenberg¹, Eric Leibert¹, Michael D Weiden¹, Harvey Pass⁴, Alexander Statnikov²^,^⁵, Kam-Meng Tchou-Wong¹^,^³, and William N Rom¹^,^³

**Online Data Supplement**

**Supplementary Methods:**

**Airway epithelial cell collection and RNA processing**. We collected peripheral airway epithelial cells by brushing the small airway of an unaffected (non-nodule) lobe, contralateral to the suspicious nodule, during bronchoscopy. A 2.0-mm cytology brush (Endo Therapy, Olympus, Center Valley, PA) was advanced distally beyond the fourth-sixth order bronchial branching, and the distal end of the brush was wedged into the distal branches with gentle gliding of the brush back and forth 10-15 times. The cytology brush was placed in an Eppendorf tube with Qiagen RNAprotect Cell Reagent (Valencia, CA). The cytology brush was spun down and the cell pellet was collected and stored at - 80° C until RNA extraction.

**MicroRNA and mRNA microarray.** We extracted total RNA using Qiagen miRNeasy Mini Kit (Valencia, CA) according to the company protocol. RNA qualitative control was performed using NanoDrop ND-1000 (Thermo Scientific, Hudson, NH) and Agilent 2100 Bioanalyzer (Agilent Technologies, Santa Clara, CA). All samples had RNA Integrity Number (RIN) greater than 6. To identify target genes in the field of cancerization, global gene expression profiling was performed with the Affymetrix^®^ (Santa Clara, CA) GeneChip Human Genome U133 Plus 2.0 Array (HG-U 133 Plus 2.0), which contains 54,000 probe sets, 47,000 genes and variants, representing ~38,500 characterized human genes. Ten µg of cRNA was hybridized to the array, and was processed with the Affymetrix system, including GeneChip Hybridization Oven 640, Fluidics Station 450 and Scanner 3000. All microarray data have been submitted to the Gene Expression Omnibus (GEO) under accession number GSE54495 (mRNA) and GSE54541 (miRNA). Reviewer accession link to GEO database: <http://www.ncbi.nlm.nih.gov/geo/query/acc.cgi?token=oxutimaizjyjfil&acc=GSE54543>

We used TaqMan Array Human MicroRNA A Cards v2.0 (Applied Biosystems, Foster City, CA), which contained 377 miRNA targets, to profile miRNA on the same RNA samples used for the peripheral airway field cancerization analysis. Five hundred ng of total RNA was used. Normalization was performed using global miRNA expression. The TaqMan Array was processed with an ABI 7900 HT (Applied Biosystems, Foster City, CA).

**Analysis of microarray mRNA and miRNA data.** Normalization of Affymetrix array data was by Robust Multichip Average (RMA) through the RMexpress software package ([http://rmaexpress.bmbolstad.com/](https://mail.nyumc.org/owa/redir.aspx?C=11e760f198944577a3ea9720054f30a8&URL=http%3a%2f%2frmaexpress.bmbolstad.com%2f)). MiRNA Taqman array data was normalized based on comparative CT representation using the global normalization method ([1](#_ENREF_1), [2](#_ENREF_2)). DAVID (Database for Annotation, Visualization and Integrated Discovery) version 6.7 (National Institute of Allergy and Infectious Disease, NIH) was used for functional enrichment analysis of mRNA Affymetrix microarray data based on the published protocol ([3](#_ENREF_3)). Gene Set Enrichment Analysis (GSEA) was used to assess concordance between our data and *a priori* defined set of genes ([4](#_ENREF_4)). DIANA-mirPath ([5](#_ENREF_5)), a web-server based pathway analysis tool, was performed on miRNA TaqMan array data. DIANA-mirPath utilizes predicted miRNA targets in coding sequences (CDS) or 3’-UTR regions provided by DIANA-microT-CDS algorithm ([6](#_ENREF_6)) or experimentally validated miRNA interactions by TarBase 6.0 ([7](#_ENREF_7)) prediction. Hierarchical heatmaps were generated with complete linkage clustering method and squared Euclidean distance measure. Integrated mRNA and miRNA selection first involved filtering for mRNA and miRNA with differential expression of FDR<0.15. Ingenuity Pathway Analysis (IPA) was used to identify top biological functions and disease/disorders, and to generate pathways regulated from integrated 49 mRNA-miRNA pairings (©2000-2013 Ingenuity Systems, <http://www.ingenuity.com>). MiRNA predicted targets for integrated analysis were generated based on TargetScan 6.2 ([8](#_ENREF_8), [9](#_ENREF_9)) prediction ([www.targetscan.org](http://www.targetscan.org)) within the IPA system. We used Support Vector Machines (SVMs) ([10](#_ENREF_10)), applied via leave-one-out cross-validation (LOOCV) protocol ([11](#_ENREF_11), [12](#_ENREF_12)) to develop molecular signatures of lung field cancerization from miRNA and mRNA data and estimate their classification performance. In order to obtain an unbiased estimate of the signature’s classification performance, all methods including identifying differentially expressed miRNA and mRNA and building classifier model, were applied by leave-one-out cross-validation protocol. In LOOCV all samples, except one, are used for selecting mRNA or miRNA (FDR<0.15 and |FC|>1.5) and training a classifier model with SVMs ([13](#_ENREF_13)). The remaining sample is used for the classifier model evaluation. The process is repeated as many times as there are samples, so that each sample can be used for the classifier model evaluation. Area under the curve generated using Mann-Whitney U statistics theory ([14](#_ENREF_14)).

**Transcriptomic data comparison to GSE44077 and GSE4115.** We obtained transcriptomic data from Kadara et al.(GSE44077) and Spira et al. (GSE4115) from the Gene Expression Omnibus (GEO) Data Sets. Each data set was extracted using GEO2R available from National Institute of Health (NIH), National Center for Biotechnology Information (NCBI) website. With regards to GSE44077, we selected samples labeled “airway distance from tumor = 5” as “farthest” or “1” as “closest” and set as cases and compared to “normal lung tissue brushing.” Only mRNAs which were significant at FDR <0.1 were selected for comparison. With regards to GSE4115, we selected samples labeled “smoker diagnosed with cancer” as cases and compared to “smoker NOT diagnosed with cancer.” Again, only mRNAs which were significant at FDR <0.1 were selected for comparison.

**Tumor Stage Comparison**. Early stage adenocarcinoma samples (stage I-II) were compared to late stage adenocarcinoma samples (stage III-IV) for differential mRNA expression using Linear Models for Microarray Data (LIMMA) with both student *t*-test *p*-value, Benjamini-Hochberg False Discovery Rate (FDR) for adjustment of multiple comparisons.

**Quantitative Real-time Reverse transcription PCR Analysis**. We isolated total RNA using Qiagen miRNeasy Mini Kit (Valencia, CA) according to the company protocol, and performed real time-PCR using Selected SYBR Green Master Mix (Applied Biosystems, Foster City, CA) and SuperScript (III First-Strand Synthesis SuperMIx for qRT-PCR) (Invitrogenon) on a 7900HT Fast Real-time PCR System (Applied Biosystems, Foster City, CA). Primers for ASCL1 (forward primer 5’-CCCAAGCAAGTCAAGCGACA-3’ and reverse primer 5’-AAGCCGCTGAAGTTGAGCC-3’), AMOTL2 (forward primer 5'- GCCAGACACTCATTCCCATT-3' and reverse primer 5'-GGCTGAATCCTCATTCTCCA-3); CLCN3 (forward primer 5’-GGAGGCAGCATTAACAGTTCT-3’ and reverse primer 5’-TCGCACCCAATCAATAGTATGGA-3’); and MAP3k8 (forward primer 5’-ATGGAGTACATGAGCACTGGA-3’ and reverse primer 5’-GCTGGCTCTTCACTTGCATAAAG-3’) were purchased from Sigma-Aldrich (St. Louis, MO). Each sample was run in triplicate. Human GAPDH (forward primer 5’-TGCACCACCAACTGCTTAGC-3’ and reverse primer 5’GGCATGGACTGTGGTCATGAG-3’) was used as a control. Relative expression was calculated using ΔΔCt method([2](#_ENREF_2)).

Individual TaqMan^®^ MicroRNA Assays (Applied Biosystems, Foster City, CA) were performed on the RNA samples of selected miRNA assays to verify the results of the Human MicroRNA Panels. TaqMan miRNA assay is based on stem-loop RT-PCR detection method, and the TaqMan probe in each assay was designed for a specific miRNA to test the mature miRNA expression. Assays analysed included miR-has-486-3p (mature miRNA sequence: CGGGGCAGCUCAGUACAGGAU), miR-has-483-5p (mature miRNA sequence: AAGACGGGAGGAAAGAAGGGAG), miR-has-374a-5p (mature miRNA sequence: UUAUAAUACAACCUGAUAAGUG), and miR-has-34c (mature miRNA sequence: AGGCAGUGUAGUUAGCUGAUUGC). RNU48 was used for miRNA internal controls. Relative expression was calculated using ΔΔCt method ([2](#_ENREF_2)). Reverse transcription was carried out using the TaqMan® MicroRNA Reverse Transcription Kit (Applied Biosystems, Foster City, CA) according to the manufacturer’s instructions. Each reaction contained 10 ng total RNA as template. Real-time PCR was performed using TaqMan Universal PCR Master Mix, No AmpErase UNG (Applied Biosystems, Foster City, CA) on a 7900HT Fast Real-time PCR System (Applied Biosystems, Foster City, CA).

**Reference**

1. Mestdagh P, Van Vlierberghe P, De Weer A, Muth D, Westermann F, Speleman F, et al. A novel and universal method for microRNA RT-qPCR data normalization. *Genome biology* 2009; 10: R64.

2. Schmittgen TD, Livak KJ. Analyzing real-time PCR data by the comparative C(T) method. *Nature protocols* 2008; 3: 1101-1108.

3. Huang da W, Sherman BT, Lempicki RA. Systematic and integrative analysis of large gene lists using DAVID bioinformatics resources. *Nature protocols* 2009; 4: 44-57.

4. Subramanian A, Tamayo P, Mootha VK, Mukherjee S, Ebert BL, Gillette MA, et al. Gene set enrichment analysis: a knowledge-based approach for interpreting genome-wide expression profiles. *Proceedings of the National Academy of Sciences of the United States of America* 2005; 102: 15545-15550.

5. Vlachos IS, Kostoulas N, Vergoulis T, Georgakilas G, Reczko M, Maragkakis M, et al. DIANA miRPath v.2.0: investigating the combinatorial effect of microRNAs in pathways. *Nucleic Acids Research* 2012; 40: W498-W504.

6. Reczko M, Maragkakis M, Alexiou P, Grosse I, Hatzigeorgiou AG. Functional microRNA targets in protein coding sequences. *Bioinformatics* 2012; 28: 771-776.

7. Vergoulis T, Vlachos IS, Alexiou P, Georgakilas G, Maragkakis M, Reczko M, et al. TarBase 6.0: capturing the exponential growth of miRNA targets with experimental support. *Nucleic Acids Research* 2012; 40: D222-D229.

8. Jan CH, Friedman RC, Ruby JG, Bartel DP. Formation, regulation and evolution of Caenorhabditis elegans 3'UTRs. *Nature* 2011; 469: 97-101.

9. Friedman RC, Farh KK-H, Burge CB, Bartel DP. Most mammalian mRNAs are conserved targets of microRNAs. *Genome Research* 2009; 19: 92-105.

10. Vapnik VN. Statistical Learning Theory. Adapitve and Learning Systems for Signal Processing, Communications, and Control Series. Wiley-Interscience; 1998.

11. Hastie T, Tibshirani R, Friedman J. *The Elements of Statistical Learning: Data Mining, Inference, and Prediction*. 9/15/2009 ed: Springer New York; 2009.

12. Simon R, Radmacher MD, Dobbin K, McShane LM. Pitfalls in the Use of DNA Microarray Data for Diagnostic and Prognostic Classification. *Journal of the National Cancer Institute* 2003; 95: 14-18.

13. Statnikov A, Wang L, Aliferis C. A comprehensive comparison of random forests and support vector machines for microarray-based cancer classification. *BMC Bioinformatics* 2008; 9: 319.

14. Hand D, Till R. A Simple Generalisation of the Area Under the ROC Curve for Multiple Class Classification Problems. *Machine Learning* 2001; 45: 171-186.
